# Design and Synthesis of SERS Materials for In Vivo Molecular Imaging and Biosensing

**DOI:** 10.1002/advs.202202051

**Published:** 2023-01-22

**Authors:** Qingqing Li, Hongqi Huo, Ying Wu, Lanlan Chen, Lichao Su, Xuan Zhang, Jibin Song, Huanghao Yang

**Affiliations:** ^1^ MOE Key Laboratory for Analytical Science of Food Safety and Biology College of Chemistry Fuzhou University Fuzhou 350108 P. R. China; ^2^ Department of Nuclear Medicine Han Dan Central Hospital Handan Hebei 056001 P. R. China

**Keywords:** biosensing, diagnosis, Raman mapping, self‐assembly, surface‐enhanced Raman scattering

## Abstract

Surface‐enhanced Raman scattering (SERS) is a feasible and ultra‐sensitive method for biomedical imaging and disease diagnosis. SERS is widely applied to in vivo imaging due to the development of functional nanoparticles encoded by Raman active molecules (SERS nanoprobes) and improvements in instruments. Herein, the recent developments in SERS active materials and their in vivo imaging and biosensing applications are overviewed. Various SERS substrates that have been successfully used for in vivo imaging are described. Then, the applications of SERS imaging in cancer detection and in vivo intraoperative guidance are summarized. The role of highly sensitive SERS biosensors in guiding the detection and prevention of diseases is discussed in detail. Moreover, its role in the identification and resection of microtumors and as a diagnostic and therapeutic platform is also reviewed. Finally, the progress and challenges associated with SERS active materials, equipment, and clinical translation are described. The present evidence suggests that SERS could be applied in clinical practice in the future.

## Graphical abstract

The development of surface‐enhanced Raman scattering (SERS) materials and their applications in imaging and biosensing in vivo are summarized. The progress of SERS application in biomedical field are highlighted, including cancer detection, identifying tumor margins for intraoperative guidance, SERS sensor for disease diagnosis, SERS‐guided multifunctional theranostic platforms, and advance in equipment and clinical trials.

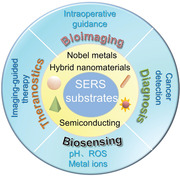

## 1 Introduction

Raman spectroscopy is vibrational spectroscopy that provides fingerprint peaks with specific characteristics.^[^ ^1^ ^]^ It was discovered in 1928 by the Indian physicist C.V. Raman who received the Nobel Prize in 1930.^[^ ^2^ ^]^ Unlike infrared spectroscopy, in Raman scattering, only one in 10^7^ photons in the target material are processed by scattering mechanisms (inelastic scattering) and exchange energy.^[^ ^3^ ^]^ Therefore, this technique is limited by its relatively low sensitivity. In 1974, Fleischmann et al. observed that the inelastic scattering of pyridine significantly increased when pyridine was attached to the silver (Ag) electrode.^[^ ^4^ ^]^ In 1977, Duyne et al. discussed the enhanced Raman signal in detail and concluded that the enhancement was associated with the increased Raman scattering efficiency of rough metal surfaces,^[^ ^5^ ^]^ called surface‐enhanced Raman scattering (SERS).^[^ ^6^ ^]^

The mechanism of SERS enhancement has not yet been fully explained, but it is usually attributed to electromagnetic (EM) and chemical (CM) enhancements.^[^ ^7^ ^]^ Among these, EM enhancement plays a major role in SERS.^[^ ^8^ ^]^ In EM enhancement, when free electrons in metal nanoparticles (NPs) are irradiated by applied light matching their electronic oscillation frequencies,^[^ ^9^ ^]^ the NPs or metal strongly absorb the photon energy in a process called local surface plasmonic resonance (LSPR).^[^ ^10^ ^]^ The EM field near the metal NPs is enhanced, especially in the gap^[^ ^11^ ^]^ called the hotspot,^[^ ^12^ ^]^ and its theoretical enhancement factors (EFs) approach ≈10^11^.^[^ ^13^ ^]^ CM enhancement is due to the CM interaction between the adsorbed molecules and the rough metal surface, inducing a charge exchange between the metal and Raman molecules.^[^ ^14^ ^]^ The resonance of Raman signals is enhanced by resonance excitation with CM EFs ranging from 10^1^ to 10^3^.^[^ ^15^ ^]^

Raman signals are increased by several orders of magnitude since molecules are usually adsorbed on the noble metal surface. Raman molecules have characteristic fingerprint peaks, conferring high sensitivity, low limit of detection (LOD), and high specificity.^[^ ^16^ ^]^ Therefore, the unique advantages of SERS have inspired extensive research in biomedical analysis and detection and early studies in cells and ex vivo tissues.^[^ ^17^ ^]^ SERS was successfully used in glucose detection in vivo in 2006^[^ ^18^ ^]^ and now it is widely used in biomedical applications owing to its high sensitivity and low background signals.

SERS imaging involves two detection methods in biomedical applications, label‐free detection and indirect detection using SERS labels.^[^ ^19^ ^]^ Label‐free detection provides the inherent fingerprint information of biomolecules with high scattering areas in biomedical samples.^[^ ^20^ ^]^ Indirect detection uses Raman molecules attached to NPs for ultrasensitive SERS detection and imaging. However, most in vivo biomolecules show a low Raman cross‐sectional area.^[^ ^21^ ^]^ Therefore, scientists usually use strong and unique Raman molecules to improve detection sensitivity. One of the main advantages of SERS is the long‐term stability of Raman molecules that generates highly reproducible SERS signals, thereby reducing the autofluorescence of the sample.^[^ ^22^ ^]^ SERS is suitable for long‐term in vivo imaging thanks to its negligible photobleaching, extremely high sensitivity, narrow fingerprint peak, and versatility. It was first applied to the non‐invasive imaging of the deep tissues of living mice in 2008.^[^ ^23^ ^]^ Multiplex imaging^[^ ^24^ ^]^ (a SERS tag with multiple marker targets) is also used for accurate disease diagnosis due to the unique and narrow Raman vibrational peaks of Raman molecules.^[^ ^25^ ^]^ More recently, SERS was used for the ultra‐sensitive detection of tiny tumor lesions, facilitating the early detection of tumors and the accurate detection of tumor margins during surgery.^[^ ^26^ ^]^

SERS has achieved serial progress in disease detection,^[^ ^27^ ^]^ multiplexing imaging,^[^ ^28^ ^]^ biomarker sensing,^[^ ^29^ ^]^ and intraoperative guidance for in vivo tumor resection.^[^ ^30^ ^]^ However, detection in human subjects requires extensive optimization of the signal intensity of SERS tags, as well as the determination of the dose and long‐term toxicity of the material, imaging penetration depth, and imaging speed.^[^ ^31^ ^]^ Like any other technology, SERS also has limitations, and related research is constantly advancing and developing. For example, ultrabright gap‐enhanced Raman tags (GERTs) with petal‐like shell structures (P‐GERTs) were reported to be excellent SERS tags.^[^ ^32^ ^]^ SERS imaging with a wide field (3.2×2.8 cm^2^) was achieved in sentinel lymph nodes within 6 s. Moreover, the combination of SERS and endoscopy was reported for the rapid imaging of the luminal surfaces of the colon and esophagus, helping to detect tiny and hard‐to‐detect lesions under white‐light endoscopy.^[^ ^33^ ^]^ In an important advancement, the combination of spatial offset Raman spectroscopy (SORS) and SERS obtained SERS signals at depths up to 45–50 mm in tissue.^[^ ^34^ ^]^ Recently, the combination of SERS and other imaging technologies has been used to exploit their respective advantages to achieve better simultaneous imaging and sensing.^[^ ^35^ ^]^ The combination of SERS with laser ablation (i.e., photothermal (PT) therapy, PTT) is used for the simultaneous detection and treatment of tumors.

**Figure 1:**
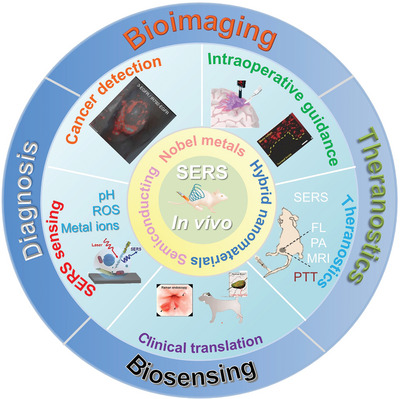
Schematic illustration of SERS active materials and their applications in biomedical fields. Intraoperative guidance image reproduced with permission.^[^ ^37^ ^]^ Copyright 2017, Wiley‐VCH. Clinical translation image reproduced with permission.^[^ ^38^ ^]^ Copyright 2019, American Chemical Society. SERS sensing image reproduced with permission.^[^ ^39^ ^]^ Copyright 2019, Wiley‐VCH. Cancer detection image reproduced with permission.^[^ ^40^ ^]^ Copyright 2015, Springer Nature.

This review provides a detailed overview of SERS as a highly sensitive and specific in vivo imaging technique. We aimed to summarize the development of SERS in in vivo imaging and biosensing based on two main related issues driving advances in SERS imaging, namely SERS probe selection and design and the optimization of instruments and techniques (**Figure** **1**). First, the commonly used SERS active materials, which range from highly SERS‐active materials such as gold (Au), and Ag, to strong plasmonic resonance nanostructures with enhanced SERS activity, including gaps,^[^ ^36^ ^]^ assemblies, and satellites, are summarized and reviewed. The hybrid nanostructures designed to enhance SERS signals and improve the biocompatibility and stability of SERS tag are also discussed. Then, the key advances in SERS for in vivo imaging in recent years are described in detail, including cancer detection, intraoperative guidance for precise tumor resection, SERS‐based sensing of the physiological environment, multimodal imaging, SERS combined therapeutic methods as diagnostic and therapeutic platforms, and the progress and challenges of SERS instrumentation and clinical translation. Finally, the development and prospects of SERS imaging in biomedicine are discussed. We hope that this review will guide the design of SERS sensing and in vivo imaging.

## 2 Design and Synthesis of SERS Tags

A SERS nanoprobe is usually composed of the following parts: 1) a plasmonic material with strong SERS activity, typically a noble metal such as Au and Ag; 2) a layer of Raman molecules with a unique and strong Raman signal; and 3) a protective layer to improve the biocompatibility and stability of the materials.^[^ ^41^ ^]^ In addition, SERS nanoprobes can be functionalized with target molecules to obtain specific detection. The design of a SERS nanoprobe is crucial to achieving strong SERS signals in biomedical field applications. Cytotoxicity and biosafety remain key factors in determining the application of SERS in clinical practice. Therefore, a suitable SERS nanoprobe should not only have a strong SERS signal but also be safe for the human body and tissues.

Plasmonic materials are the core of SERS nanoprobes and produce an enhanced electric field under selected laser excitation to amplify Raman signals, and their CM composition, morphology, and size significantly influence the performance of SERS nanoprobes.^[^ ^42^ ^]^ Recently, various plasmonic nanomaterials, such as nanospheres, nanorods, nanocages, nano triangles, nanostars, and nanopyramids, have been developed to meet in vivo imaging needs.^[^ ^14^ ^]^ The optical properties of SERS active nanomaterials and the design and synthesis of SERS sensors are described in detail in the following sections and **Table** **1**.

**Table 1:** Classification of SERS active materials

Category	Type	Components	Ref.
Noble metals	Gold (Au)	AuNPs	[11, 40, 43]
	Sliver (Ag)	Ag@SiO_2_ NPs	[44]
Semiconductors	Cu_2_O, TiO_2_	CuS, Cu_2_O	[45]
	Bimetals	Ag@Au	[46]
	Graphene‐metal NPs	Au@GO NP, GO‐Au@PAN, graphene/Cu	[47]
Hybrid nanomaterials	Au and carbon nanotubes	SWCNT	[48]
	Black phosphorus/metal	BP‐AuNPs	[49]
		Ag/BP‐NS	[50]
	Au‐topological insulator	Bi_2_Se_3_@Au	[51]
	Biohybrid NPs	Au@Tat peptide	[52]
Morphology	Nanospheres	AuNPs, AgNPs	[53]
	Nanorods	AuNRs	[54]
	Nanostars	AuNSts	[55]
	Nanobipyramids	AuNBps	[56]
	Triangular nanoplates	Au@Ag nanoplates	[46b]
	Nanowire	AgNWs	[57]
	Core‐shell	Ag/SiO_2_, Au@Ag, Ag@Au, Au@Cu_2−x_S	[44, 58]
	Nanogap	Nanogap Au@Au	[59]
Assembled nanostructures	Core‐satellite	AuNR‐AuNP, AuNNR@MSN@AuNPs, Au@Ag@SiO_2_‐AuNP	[60]
	Sandwich structure	AuNP‐decorated Ag@SiO_2_ nanocomposite, Carbon nanodot‐decorated Ag@SiO_2_	[61]
	Au nanowire	Nanogap‐rich Au NWs	[62]
	AuNP chain nanopeapod	Gap‐separated linear chain AuNP‐SNTPs	[63]
	Vesicles	AuNP vesicles, AuNR vesicles	[64]

### 2.1 Gold‐Based Nanomaterials

Gold nanoparticles (AuNPs) are the most widely studied SERS active materials in in vivo imaging since they display less toxicity, higher biocompatibility, and greater stability than silver nanoparticles (AgNPs).^[^ ^70^ ^]^ More importantly, Au plasmonic nanostructures show LSPR in the near‐infrared (NIR) region by changing their structure, shape, or size. Thus, more studies have focused on Au‐based materials.^[^ ^71^ ^]^ Plasmonic nanostructures of different sizes and shapes are widely studied in chemical synthesis to produce stronger SERS signals.^[^ ^72^ ^]^ The assembled plasmonic nanostructures are composed of multiple NPs with excellent SERS properties and are prepared by electrostatic adsorption,^[^ ^73^ ^]^ covalent binding,^[^ ^60^, ^74^ ^]^ and hydrophobic interactions.^[^ ^64^, ^75^ ^]^ This section reviews the properties of different types of Au‐based nanomaterials.

#### 2.1.1 Gold Nanocrystals

**Figure 2:**
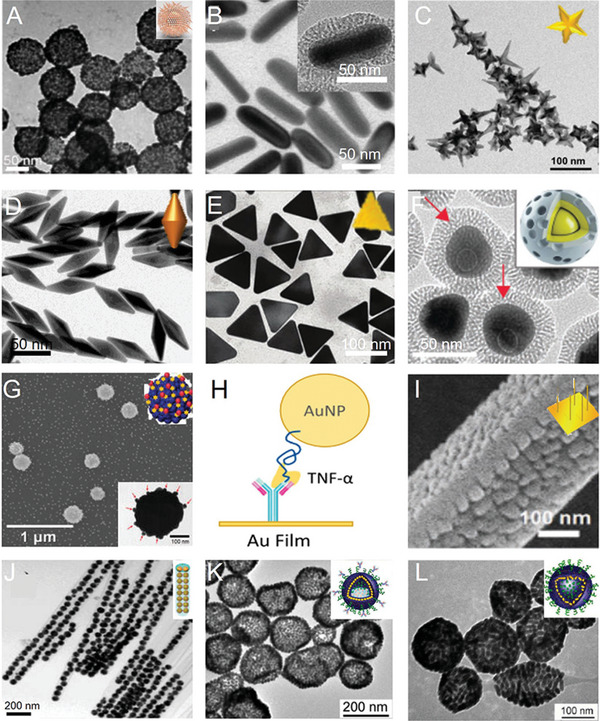
Gold nanocrystals and their assemblies. A) TEM image of branched nanoporous gold nanoshells. Reproduced with permission.^[^ ^65^ ^]^ Copyright 2017, American Chemical Society. B) TEM image of gold nanorods. Reproduced with permission.^[^ ^66^ ^]^ Copyright 2017, American Chemical Society. C) TEM images of gold nanostars. Reproduced with permission.^[^ ^67^ ^]^ Copyright 2018, The Royal Society of Chemistry. D) TEM image of gold nanobipyramids. Reproduced with permission.^[^ ^56a^ ^]^ Copyright 2017, American Chemical Society. E) TEM image of triangular nanoplates. Reproduced with permission.^[^ ^46b^ ^]^ Copyright 2018, The Royal Society of Chemistry. F) TEM image of gap‐enhanced Raman tags. Reproduced with permission.^[^ ^68^ ^]^ Copyright 2018, Wiley‐VCH. G) SEM image of Ag shell‐Au core‐satellite nanostructure. Reproduced with permission.^[^ ^46a^ ^]^ Copyright 2014, American Chemical Society. H) Schematic of the formation process of the sandwich nanostructure. Reproduced with permission.^[^ ^61a^ ^]^ Copyright 2017, Proceedings of the National Academy of Sciences of the United States of America. I) TEM image of nanogap‐rich Au nanowire. Reproduced with permission.^[^ ^62^ ^]^ Copyright 2017, Wiley‐VCH. J) TEM image of gap‐separated linear chain gold NP‐embedded silica nanotube peapods (SNTPs). Reproduced with permission.^[^ ^63^ ^]^ Copyright 2014, American Chemical Society. K) TEM image of gold NP vesicles. Reproduced with permission.^[^ ^64a^ ^]^ Copyright 2012, American Chemical Society. L) TEM image of gold nanorod vesicles. Reproduced with permission.^[^ ^69^ ^]^ Copyright 2013, American Chemical Society.

Gold nanospheres (AuNsps) are the common core of SERS tags^[^ ^53a^ ^]^ since they have a stable structure, simple synthetic procedure, and can be easily modified into different structures (**Figure** **2**A).^[^ ^53a^ ^]^ AuNPs are prepared by reducing chloroauric acid with sodium citrate or seed‐mediated methods with boiling. The LSPR peak of AuNsps is approximately 520 nm and shows a limited red‐shift trend with increases in NP size. The SERS performance of Au core‐shell nanospheres can be adjusted by changing the size of the core, composition, thickness, and shape of the shell.^[^ ^76^ ^]^ Song et al. developed a branched nanoporous gold nanoshell (BAuNSP) with adjustable surface roughness and nano‐gap using redox‐active poly(vinyl phenol)‐b‐(styrene) as a template and reducing agent.^[^ ^65^ ^]^ A large number of hotspots were presented on the nanoporous and sharp branches of the BAuNSP surface, generating an enhanced EM field and improving the optical properties. The SERS signal and PT effect of BAuNSP were significantly improved, and it was a good SERS nanoprobe for in vivo imaging.

A significant advantage of gold nanorods (AuNRs)^[^ ^54^ ^]^ (Figure 2B) is their tunable longitudinal plasmonic resonance, which can be adjusted by varying the aspect ratio (length‐to‐width).^[^ ^77^ ^]^ AuNRs are prepared under the action of surfactants and shape inducers by the seed‐mediated method. The LSPR peaks of AuNRs shift from the visible region to the NIR region with increasing AuNR aspect ratios. The Raman‐enhanced effect of AuNRs depends on their aspect ratio and size. A series of plasmonic nanomaterials based on AuNRs were developed in the range of the first NIR (NIR‐I) to the second NIR (NIR‐II) region.^[^ ^78^ ^]^ A wider surface plasmonic resonance (SPR) tunability of core‐shell AuNRs was achieved by changing the core size or shell material.^[^ ^79^ ^]^ Ye et al. reported an ultrabright SERS nanoprobe with metallic Au@Ag core‐shell rod‐like nanostructures (RMNs) and embedded Raman molecules. Strong SERS enhancement and tunable PT were achieved by adjusting the Ag shell thickness.^[^ ^66^ ^]^

Gold nanostars (AuNSts) (Figure 2C) are an important type of Au nanostructures, successfully used in in vivo tumor detection by SERS imaging.^[^ ^80^ ^]^ AuNSts not only display tunable LSPR in the NIR window,^[^ ^55a^ ^]^ but also their sharp metal tip produces strong SERS enhancement and high PT conversion efficiency.^[^ ^58^, ^80^ ^]^ Wu et al. developed Ramon molecules with 3,3′‐diethylthiatricarbocyanine iodide (DTTC)‐conjugated Ag_core_@Au_shell_ nanostars to achieve enhanced in vivo SERS imaging and the PTT of tumors.^[^ ^81^ ^]^ The SERS signal was significantly enhanced and the biocompatibility was improved due to the Au nanostar coating on the AgNPs. The Ag_core_@Au_shell_ nanostars demonstrated high PT conversion efficiency, and the tumors almost completely disappeared during 14 days of treatment.

Gold nanobipyramids (AuNBPs) (Figure 2D) have stronger local electric field enhancement than nanorods or other nanostructures due to their two sharper tips. AuNBPs exhibit enhanced SERS signals and are widely studied in SERS imaging. Zhou et al. successfully synthesized an AuNBP probe conjugated with a Raman molecule and folic acid, which was useful for the quantitative detection of MCF‐7 cancer cells (5–500 cells mL^−1^) and the enhancement of Raman signals in vitro and in vivo.^[^ ^56a^ ^]^

The structure of gold triangular nanoplates (AuTNPs) is characterized by a sharp apex and a large specific surface (Figure 2E). AuTNPs show adjustable LSPR peaks from the visible region to the NIR region, while the sharp tips and corners lead to greater local EM field enhancement, making them suitable for in vivo imaging. Nie and colleagues successfully developed Au@Ag triangular nanoplates by coating thin Ag layers onto Au triangular nanoplate surfaces. The Raman signal and optical absorption of the Ag‐hybridized plasmonic Au‐triangular nanoplates were stronger than that of AuNPs due to the greater Ag contribution to the enhancement of the electric field.^[^ ^46b^ ^]^ This unique core‐shell structure showed great potential as a multi‐diagnostic platform for cancer detection and treatment.^[^ ^82^ ^]^

Gap‐enhanced core‐shell structures (Figure 2F),^[^ ^68^ ^]^ also known as nanomatryoshkas, generate a large number of EM and CM hotspots in the gap,^[^ ^11^, ^83^ ^]^ resulting in strong SERS performance and ultra‐high sensitivity.^[^ ^84^ ^]^ Various gap‐enhanced core‐shell structures for in vivo applications have been reported, and Raman molecules have been placed in gap junctions,^[^ ^59^, ^85^ ^]^ significantly improving the SERS signal and protecting Raman active molecules from adverse environments, such as in circulating blood and the tumor microenvironment.^[^ ^86^ ^]^ Thus, they have a highly stable signal and are used in the accurate diagnosis of microsatellite tumors.^[^ ^87^ ^]^

#### 2.1.2 Gold NP Assembly

Core‐satellite assemblies consist of a metallic core and some small metallic NPs as satellites (Figure 2G).^[^ ^88^ ^]^ They are usually prepared by electrostatic adsorption,^[^ ^89^ ^]^ covalent bonding,^[^ ^60c^ ^]^ and DNA hybridization.^[^ ^88^, ^90^ ^]^ They exhibit strongly coupled plasma and are a perfect example of a SERS nanoprobe.^[^ ^91^ ^]^ Core‐satellite nanostructures generate hotspots from the nanogap junctions of the NPs, where EM fields are significantly concentrated, allowing ultra‐sensitive SERS signals. The core‐satellite showed enhanced plasmon resonance compared to single AuNPs, broadening their applications in biomedicine.^[^ ^41^ ^]^ Jeong et al. successfully prepared a heterostructure Ag shell‐Au satellite (Ag‐Au SS), presenting strong and highly homogeneous SERS activity.^[^ ^46a^ ^]^

The sandwich structure with an enhanced local EM field at the interlayer created an effective SERS hotspot, generating strong SERS signals (Figure 2H).^[^ ^61b^ ^]^ Moskovits et al. successfully fabricated an AuNP‐Au film sandwich structure with a Raman‐labeled affinity reagent to connect AuNPs and another Raman‐labeled affinity reagent to connect the Au film.^[^ ^61a^ ^]^ SERS intensity was enhanced from the two Raman reporter molecules in the hotspot, resulting in the specific detection of *α*‐thrombin in human serum, with a LOD of 86 pm.

The plasma structure of nanogap‐rich Au nanowire (AuNW) is comprised of many regularly distributed AuNPs on an AuNW with uniformly distributed nanogaps,^[^ ^92^ ^]^ creating a large number of hotspots between adjacent nanogaps and strong Raman signals.^[^ ^93^ ^]^ Kim and colleagues successfully prepared a nanogap‐rich AuNW SERS sensor with multiple and uniformly distributed nanogaps (Figure 2I) to detect telomerase activity in cancer cells (LOD of 0.2 cells mL^−1^) and accurately diagnose gastric and breast cancers.^[^ ^62^ ^]^

Linear silica nanotube pods (SNTPs) (Figure 2J) are fabricated by self‐assembly, with a 1 nm nanogap between internal AuNPs.^[^ ^63^ ^]^ SNTPs exhibited excellent SERS signals due to the well‐maintained nanogap junction, and the protection of the silica wall resulted in good SERS in vivo imaging.

Self‐assembled plasmonic nanostructures^[^ ^94^ ^]^ generated enhanced local electric fields at interparticle NPs where Raman molecules were placed, resulting in a strong SERS signal.^[^ ^60^, ^75^ ^]^ Song et al. fabricated SERS‐coded plasma vesicles via the self‐assembly of amphiphilic polymer brush‐coated AuNPs, showing significantly enhanced SERS intensity due to strong plasma coupling (Figure 2K).^[^ ^64a^ ^]^ They also constructed a bio‐conjugated SERS‐active self‐assembled vesicle structure based on an AuNR assembly (Figure 2L), showing a large number of hotspots between strongly coupled AuNRs, effectively generating highly active and repeatable SERS signals and sensitively detecting cancer cells.^[^ ^69^ ^]^ A recent study described the design of probes, such as Au or Ag plasmon NPs self‐assembled into SERS nanoclusters in living cells, which targeted surface biomarkers in cancer cells.^[^ ^95^ ^]^ Selective and sensitive SERS imaging of the target cells was achieved due to the near‐field amplification of Raman fingerprints within the plasma hotspots.

In summary, self‐assembled nanomaterials enhance local EM fields by their aggregation and are also used in bioimaging and detection.^[^ ^53c^ ^]^ The self‐assembly process is used to prepare NPs with different sizes, shapes, compositions, and nanogaps to adjust to the corresponding SPR. When NP spacing is reduced, the SERS response is greatly enhanced, resulting in high sensitivity in vivo imaging and detection.^[^ ^60^, ^96^ ^]^

### 2.2 Ag Nanoparticles

**Figure 3:**
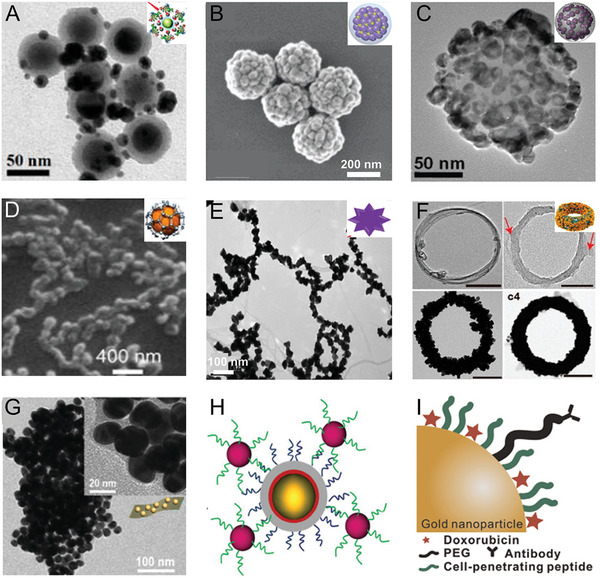
A) TEM image of dual‐mode fluorescence‐SERS tags (UCNP@SiO_2_@Ag nanocomposites). Reproduced with permission.^[^ ^97^ ^]^ Copyright 2014, American Chemical Society. B) TEM image of silica‐coated Ag bumpy nanoshell (AgNS@SiO_2_). Reproduced with permission.^[^ ^98^ ^]^ Copyright 2017, The Royal Society of Chemistry. C) TEM image of Au/Ag hollow‐shell NP. Reproduced with permission.^[^ ^99^ ^]^ Copyright 2017, Wiley‐VCH. D) TEM image of graphene/CuNP. Reproduced with permission.^[^ ^47c^ ^]^ Copyright 2015, American Chemical Society. E) TEM image of Au nanopopcorn‐coated SWCNT. Reproduced with permission.^[^ ^100^ ^]^ Copyright 2011, American Chemical Society. F) TEM image of CNT ring coated with AuNPs. Reproduced with permission.^[^ ^101^ ^]^ Copyright 2016, American Chemical Society. G) TEM image of hybrid black phosphorus‐AuNPs. Reproduced with permission.^[^ ^49^ ^]^ Copyright 2018, The Royal Society of Chemistry. H) Schematic of targeted SERS nanoprobes. Reproduced with permission.^[^ ^102^ ^]^ Copyright 2017, Wiley‐VCH). I) Schematic of a hybrid nanoparticle. Reproduced with permission.^[^ ^52^ ^]^ Copyright 2015, Elsevier.

Ag is a more effective SERS active material than Au, with a higher SERS EF due to its large scattering area, and it has a lower price than Au. However, it has short stability, poor biocompatibility, and uncontrollable size compared to Au, limiting its in vivo applications.^[^ ^103^ ^]^ Therefore, the surface of an Ag‐based SERS sensor is usually covered with a protective layer (e.g., bovine serum albumin (BSA),^[^ ^98^ ^]^ silica encapsulation, polyethylene glycol (PEG) coating, or an Au shell^[^ ^81^ ^]^) to reduce the toxicity of AgNPs. Moreover, shell coating avoids aggregation, and improves the stability and dispersion of AgNPs.^[^ ^104^ ^]^ An up‐conversion fluorescence‐SERS dual‐mode probe (UCNP@SiO_2_@Ag) was developed using AgNPs grown in situ on SiO_2_‐coated UCNPs to enhance the Raman signals and BSA coating on the Ag surface to improve the stability and biocompatibility of the nanoprobe (**Figure** **3**A).^[^ ^97^ ^]^ Importantly, silica‐coated Ag NPs displayed high dispersibility, high stability, easy surface function, and excellent biocompatibility. Dae et al. prepared silica‐coated Ag bumpy nanoshell probes (AgNS@SiO_2_) with strong SERS activity and high NIR absorption, showing great potential in the multiplexed detection of biomarkers (Figure 3B).^[^ ^98^ ^]^

### 2.3 Hybrid Nanomaterials

Hybrid materials, such as bimetals^[^ ^79^ ^]^ and graphene/Cu NP hybrids,^[^ ^47^, ^105^ ^]^ have also been extensively studied as the combined properties of different nanomaterials showed lower toxicity, improved biocompatibility, and adjustable plasmonic properties over a long wavelength range.^[^ ^106^ ^]^ These properties broaden the applications of nanomaterials in bioimaging.

#### 2.3.1 Bimetals

The most common combination is Au and Ag, using the strong SERS EF of Ag and good biocompatibility of the Au shell. This combination produces strong SERS signals and reduces toxicity, extending their applications in vivo. Recently, Au‐Ag core‐shell nanostructures such as Ag@Au nanocubes and Ag@Au nanostars were fabricated for in vivo SERS imaging.^[^ ^27b^ ^]^ Ag@Au nanostars were designed for NIR‐triggered highly efficient PTT, and they exhibited excellent SERS enhancement performance and PT conversion efficiency due to the metal tip structure.^[^ ^81^ ^]^ Lee et al. synthesized Au/Ag hollow shell (HS) plasmonic nanostructure silica nanospheres (Figure 3C) with an inner hollow diameter adjustable from 3 to 11 nm by changing the Au^3+^ ratio. This property allowed the red‐shifting of the plasmonic extinction of Au/Ag NPs into the NIR range (650–900 nm), generating a stronger SERS signal than bare spherical AuNPs.^[^ ^99^ ^]^

#### 2.3.2 Carbon‐Based Nanomaterials Coupled with Metals

Carbon nanotubes (CNTs)^[^ ^107^ ^]^ and graphene (rGO)^[^ ^108^ ^]^ show typical Raman characteristic peaks and are often used as SERS sensors for biomedical applications.^[^ ^109^ ^]^ rGO is a 2D atomic crystal with dense carbon atoms in the crystal lattice,^[^ ^108a^ ^]^ creating space for the charge transfer between the rGO surface and adsorbed molecules, resulting in enhanced Raman signals.^[^ ^110^ ^]^ The enhancement is defined as rGO‐enhanced Raman scattering (GERS). Carbon‐based material can also be coupled with Au, Ag, or other SERS active metals, which helps to enhance their SERS signals.^[^ ^47b^ ^]^ When single‐layer rGO was grown directly on a CuNP surface to prepare a high‐performance SERS substrate, rGO covered each part of the rGO/Cu hybrid NPs (Figure 3D).^[^ ^47c^ ^]^ The target molecule was efficiently adsorbed into the gaps and adenosine in the serum was detected at a LOD of 5 nm.

Single‐walled carbon nanotubes (SWNTS) (Figure 3E) can effectively produce strong Raman signals due to their strong electron‐phonon coupling.^[^ ^23^ ^]^ Hybrid rGO/CNT and Au NPs are considered strong SERS substrates.^[^ ^100^ ^]^ Raman signals are significantly enhanced when CNTs are placed among AuNPs, and AuNPs serve as local nanoantennas, with corresponding increases in plasmonic resonance. Song et al. designed a new carbon nanotube ring (CNTR) coated with AuNPs (CNTR@AuNPs) to enhance the plasma coupling of AuNP. The SERS signal was approximately 110 times higher than that of CNTR (Figure 3F).^[^ ^101^ ^]^ CNTR embedded in AuNP gaps was used as a Raman probe and photoacoustic (PA) contrast for imaging and imaging‐guided cancer therapy in two tumor models.

Different nanostructures based on CNT, rGO, and self‐assembled with metal particles have been studied^[^ ^111^ ^]^ and displayed enhanced SERS performance and good biocompatibility. Thus, they can be used as SERS substrates for in vivo SERS imaging.

#### 2.3.3 Black Phosphorus‐Based Nanomaterials Coupled with Metals

Black phosphorus (BP) is a 2D nanomaterial with a high surface area, which can generate unique Raman signals. However, its intrinsic Raman signal is weak. Hybrid SERS substrates combining metal NPs with BP exhibited enhanced SERS activity, which could be ascribed to the EM enhancement caused by a large number of hotspots in the gaps between metal NPs and the additional CM enhancements originating from the charge transfer between BP and the analyte molecule.^[^ ^112^ ^]^ Guo et al. successfully fabricated black phosphorus‐AuNP hybrids (BP‐AuNPs), where AuNP deposition on BP nanosheets provided excellent NIR SERS activity, whereas BP‐AuNPs had a lower Raman background signal than rGO‐based layered platforms (Figure 3G).^[^ ^49^ ^]^ BP‐AuNPs produced high bio‐SERS signals owing to the Raman enhancement effects of BP and AuNPs and the low background of BP.

Overall, a variety of hybrid nanomaterials, such as rGO/Cu hybrids,^[^ ^47c^ ^]^ plasmonic Au‐Ag hollow‐shell assemblies,^[^ ^99^ ^]^ and biohybrid NPs, have been created for in vivo SERS imaging.^[^ ^52^ ^]^ These hybrid nanomaterials not only have outstanding biocompatibility, but also significantly expanded SERS applications.^[^ ^113^ ^]^

### 2.4 Modification of SERS Tags

SERS tags for in vivo imaging usually consist of Raman‐enhanced NPs and Raman‐active molecules. Generally, SERS NPs passively target the tumor due to the enhanced permeability and retention (EPR) effect.^[^ ^19^ ^]^ The most important aspect of a SERS label is that it should have good biocompatibility, and it is usually encapsulated to protect Raman molecule activity.^[^ ^114^ ^]^ Polymers such as PEG,^[^ ^115^ ^]^ polystyrene,^[^ ^116^ ^]^ and encapsulated silica layers are most commonly used to modify SERS NPs, ensuring the activity of Raman molecules, and are beneficial to the functionalization of SERS tags.

SERS nanoprobes can be functionalized with antibodies, aptamers, DNA,^[^ ^26^ ^]^ peptides, or folic acid^[^ ^102^ ^]^ for the sensitive detection of tumors and active targeting of specific tumor regions (Figure 3H).^[^ ^102^ ^]^ For example, aptamers are used as biomarkers targeting cancer. Kircher et al. designed a functionalized SERS nanoprobe with DNA aptamers that specifically targeted mucin1 (MUC1), which is overexpressed in breast cancer.^[^ ^102^ ^]^ The injection of non‐targeted or targeted SERS NPs on the left and right side of the same mouse, respectively, demonstrated that the active homing of the tumor by targeted SERS NPs resulted in greater accumulation than that of passive/EPR‐targeted NPs. Choi et al. constructed a biocompatible hybrid consisting of AuNPs, cell‐penetrating peptide (Tat peptide), cancer‐targeting antibody, and doxorubicin (DOX)‐enhanced SERS signal (Figure 3I).^[^ ^52^ ^]^ In this way, the biocompatible hybrid could specifically target HER2‐positive cancer cells (SK‐BR‐3). Biohybrid NPs loaded with DOX were specifically immobilized on the target cell membranes to rapidly penetrate the cells, and DOX was released when it encountered glutathione (GSH). Therefore, drug release was monitored by time‐dependent SERS signal changes.

## 3 Application of SERS Imaging In Vivo

Bioimaging, involving tracking biomarkers and visualizing specific biological processes, is of great significance in biomedical research. SERS has received extensive attention in in vivo imaging due to its excellent sensitivity, high spectral resolution for multiple detections, and excellent resistance to photobleaching. SERS imaging was first applied to in vivo detection in 2006,^[^ ^18^, ^117^ ^]^ and has been increasingly used for in vivo imaging and biosensing in recent years. In the following sections, the recent advances in in vivo imaging using SERS nanoprobes over the past decade are discussed **Table** **2**.

**Table 2:** SERS nanoprobes in biomedical applications

Classification	Application	SERS tags	Advancements	Ref.
Bioimaging	Tumor detection	AuNP‐reporter‐PEG	Tumor‐targeting detection	[118]
		AuNP‐dye@SiO_2_ shell	With attomolar LOD^a)^	[40, 102]
	Multiplex imaging	AuNP‐reporter‐PEG	10 different spectral fingerprints	[25a]
		AuNP‐dye@SiO_2_ shell	Five‐plex ratiometric imaging of tumors	[119]
	Ultrabright and high‐speed bioimaging	AuNNP‐ reporter @petal shell	3.2 × 2.8 cm^2^ area imaging within 52 s	[32a]
Intraoperative guidance	Identifying tumor margins	AuNSt‐dye@SiO_2_‐*α*FR‐Ab	Detected 370 µm tumors	[26]
	Guided tumor resection	AuNP‐reporter@SiO_2_ shell	Delineation of cancerous lesion margins without specific biomarker targeting	[30b]
		Au‐Ur@DTTC NPs	Observed invisible margins and residual tumor	[120]
	Using a handheld Raman scanner	AuNP‐reporter@SiO_2_ shell	Detected microscopic lesions not seen on static SERS images	[121]
	Eliminating residual microscopic lesions	Gap Au‐reporter@Au core–shell	Detection of residual microtumors and ablation of malignant lesions	[86]
		AuNSt‐reporter@SiO_2_	SERS‐guided thermosurgery to eliminate residual microtumors	[55b]
Biosensing	pH	Au‐QT‐reporters	Simultaneous quantification of CO_3_^2‐^ and pH in live brains and neurons	[39]
		AuNSt‐IR7p	Ratiometric SERS imaging for delineation of tumor acidic margin and guided surgery	[122]
	ROS	AuNNR‐reporter 1@Au@MSi‐AuNP‐ reporter 2	Ratiometric SERS quantitative detection of H_2_O_2_ in vivo	[60c]
	Metal ions	AuNPs@QT‐reporter	Simultaneous quantification of Cu^+^ and Cu^2+^ concentrations in the cerebral cortex in vivo	[123]
	Glucose	AuNPs@MIL‐101 @oxidases	Monitoring of changes in glucose and lactate	[124]
	Nucleic acid biomarker	AuNW@AuNPs‐TB	Detection of telomerase activity and diagnosis of gastric and breast cancer	[62]
Multifunctional platform	Dual‐modality imaging	AuNSt‐reporter@SiO_2_	Dual‐modality SERS and PA imaging	[125]
		AuNR@dye‐DNA@SiO_2_	Fluorescence‐Raman bimodal imaging	[126]
		Au@Prussian blue‐Gd@ovalbumin	MR/SERS for tracking DC migration	[127]
	Multimodal imaging	AuNP‐reporter@SiO_2_@Gd	MRI‐PA‐SERS NPs for tumor resection guidance	[128]
		AuFeNP@reporter	SERS‐MRI‐CT Imaging^b)^	[129]
		AuNP‐dye@SiO_2_@^68^Ga	SERS‐PET‐MR for lymph node tracking1^c)^	[130]
	Theranostic platforms	AuNP‐reporter@Cu_2−x_S core‐shell	SERS imaging‐guided PTT	[45b]
		AuNFs‐reporter‐Dox	SERS and PA‐guided photo‐chemotherapy	[131]
		Au@GO NP‐NACs	SERS‐guided synergism of photothermal, genetic, and chemotherapy	[47a]
Progress in instrumentation and translation	Preclinical endoscopic imaging	AuNP‐reporter@SiO_2_	SERS endoscopy for the detection of premalignant gastrointestinal lesions	[38b]
	Handheld Raman detector combined with optical clearing agent	AuNSt‐reporter@PEG shell	Depth of detection from superficial tissues to subcutaneous or deeper lesions masked by the dermal tissue	[132]
	SESORS	Chalcogenpyrylium reporters	Depth of detection up to 25 mm	[133]
		AuNSt‐reporter@SiO_2_‐RGDyK peptide	Obtained Raman spectra of deep GBMs in mice	[134]
	Clinical trial	AuNP‐reporter@SiO_2_‐PEG	Nanoparticle‐based EPR for spontaneous canine brain tumors	[38a]
		AuNP‐reporter@SiO_2_‐mAb	Rapid (< 15 min) detection of surgical margins	[135]

^a)^ Abbreviations: LOD, limit of detection

^b)^ MRI, magnetic resonance imaging; CT, computed tomography

^c)^ PET, positron emission tomography.

### 3.1 SERS Imaging for Cancer Detection and Intraoperative Guidance

#### 3.1.1 SERS Imaging for Tumor Detection and Microscopic Tumor Identification

**Figure 4:**
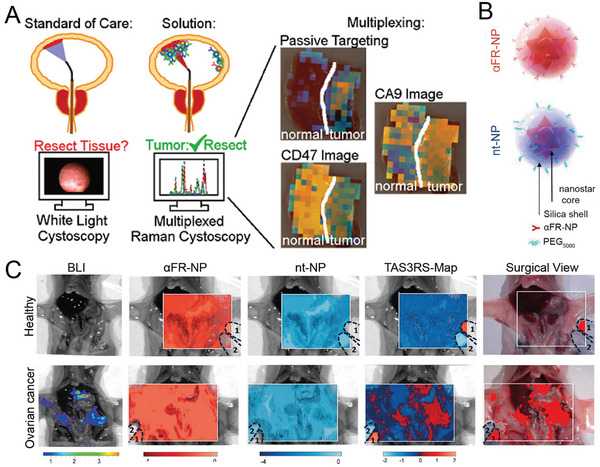
A) Applications of intraluminal SERS NPs. Different targeting mechanisms correspond to different colored NPs (CA9, red; passive, blue; CD47, green). Reproduced with permission.^[^ ^136^ ^]^ Copyright 2018, American Chemical Society. B) Schematic diagram of the composition of SERS nanoprobes. The Au nanostar was grafted with Raman molecules IR780 (red) or IR140 (blue), which were then coated with a silica shell. They were then modified with folate receptor‐targeting antibodies (*α*FR‐Ab) or PEG to prepare targeted NPs (*α*FR‐NPs, red) and non‐targeted NPs (nt‐NPs, blue), respectively. C) SERS imaging of the whole abdomen of healthy mice (left) and tumor‐bearing mice (right). Reproduced with permission.^[^ ^26^ ^]^ Copyright 2017, American Chemical Society.

Recently, SERS imaging has been extensively used for tumor detection. SERS NPs accumulate in tumors for in vivo imaging by two main mechanisms, active targeting, and passive targeting.^[^ ^137^ ^]^ SERS, with characteristic fingerprint peaks, is suitable for the detection of multiple biomarkers, which helps to improve detection accuracy, offering enough information for disease diagnosis.^[^ ^138^ ^]^ Zavaleta et al. successfully constructed 10 unique SERS NPs for the skin of living mice to demonstrate highly non‐invasive multiplexing.^[^ ^25a^ ^]^ Gambhir et al. evaluated the accuracy of the active targeting of antibody‐modified SERS NPs and the passive targeting of the tumor site by topically applying SERS NPs to seven normal bladder tissues and eight bladder tumor tissues (**Figure** **4**A).^[^ ^136^ ^]^ An active‐to‐sum normalization method was proposed using actively targeted SERS NPs (CD47 and CA9) and passively targeted SERS NPs, which normalized each channel to the sum of all three channels. The comparison of passively targeted NP imaging with active‐to‐sum standardized multiple imaging methods found that active‐passive normalization showed a lower background signal and higher detection accuracy. The molecular approach showed promising potential for distinguishing tissue injury from cancer, reducing the time physicians spend resecting inflamed tissue.

Local recurrence and metastasis are mainly caused by malignant cells that are not completely removed during surgery.^[^ ^139^ ^]^ Some studies showed that SERS imaging could detect microscopic tumors, which were invisible under a white light microscope.^[^ ^45a^ ^]^ SERS imaging is expected to detect microscopic tumor lesions and realize more complete tumor resection.^[^ ^140^ ^]^ Kircher et al. demonstrated that^[^ ^139^ ^]^ SERS imaging not only allowed a clear recognition of tumor margins but also detected numerous tiny cancerous lesions that were not visible under magnetic resonance imaging (MRI) or white light exposure using silica‐encapsulated SERS NPs. Furthermore, integrin‐targeted SERS nanoprobes (RGD‐SERS) could detect tiny tumor areas far from the main tumors and accurately identify tiny metastases microscopically and independent cancer cell clusters of no more than five cells in glioblastoma.^[^ ^141^ ^]^ Ovarian cancer was detected using a ratiometric method of folic acid‐targeting SERS NPs and non‐targeting SERS NPs (Figure 4B).^[^ ^26^ ^]^ The ratiometric method has the potential to detect tiny residual tumors during surgery, and bioluminescence imaging (BLI) and histological staining confirmed that tumors of 370 µm in size were detectable (Figure 4C).

#### 3.1.2 Intraoperative Guidance of Tumor Resection

**Figure 5:**
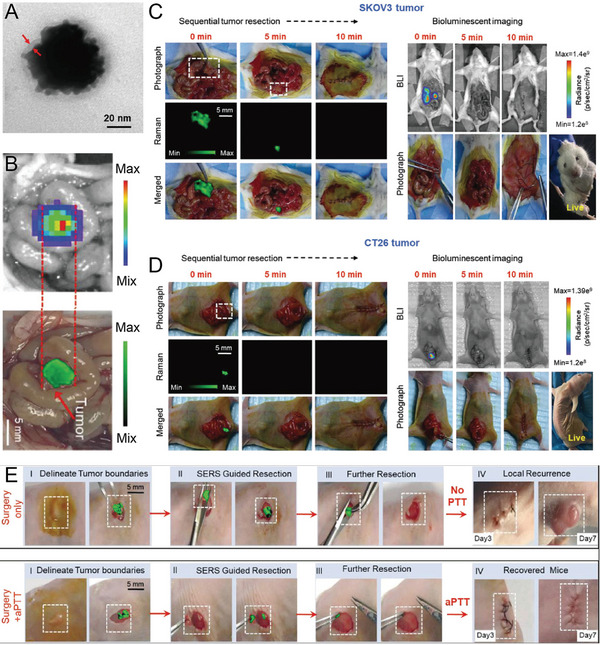
A) TEM image of urchin‐like Au‐Ur@DTTC nanoparticle. B) Overlay images of photograph with bioluminescent images and Raman mapping of orthotopic CT26 colon tumor‐bearing mice. C) Abdominal SERS and BLI images of the mice during the step‐by‐step resection surgery of an SKOV3 ovarian tumor. D) SERS and BLI images during Raman image‐guided resection of an orthotopic CT26 tumor. E) Raman image‐guided subcutaneous SKOV3 ovarian tumor resection and combined PTT for the treatment of residual tumors. Reproduced with permission.^[^ ^120^ ^]^ Copyright 2021, Wiley‐VCH.

The partial resection of a tumor is a common surgical procedure used in cancer treatment, in which the complete resection of the primary tumor reduces the recurrence rate.^[^ ^142^ ^]^ No suitable method to detect the positive margin of incomplete tumor resection has resulted from clinical trials, and as many as 20%–55% of patients undergo secondary surgery and post‐radiation therapy.^[^ ^143^ ^]^ Recent studies demonstrated that intraoperative SERS imaging could accurately identify tumor margins to guide surgery^[^ ^144^ ^]^ A typical example is the use of SERS spectroscopy for detecting breast cancer.^[^ ^117^ ^]^ Data analysis and pathological examination revealed that Raman spectroscopy could detect the positive edge of breast cancer, guiding the resection of the tissue and avoiding a second surgery. Recently, Zhou et al. synthesized urchin‐like AuNPs for SERS imaging‐guided tumor resection and the PT ablation of residual tiny tumors (**Figure** **5**A).^[^ ^120^ ^]^ In situ Raman imaging of orthotopic CT26 colon tumors in mice was consistent with the BLI results of the tumor lesions, with almost no SERS signal in the surrounding normal tissues, indicating that SERS imaging could sensitively delineate the edge of the tumor (Figure 5B). These enhanced Raman signals were used to visualize and guide the removal of invisible tumor residue in subcutaneous and in situ ovarian and colon tumors until no other SERS signals were detected in the tumor bed (Figure 5C,D). The residual tumor on the edge of ovarian cancer in mice was successfully ablated by the postoperative PTT, thereby increasing the survival rate by 75% and delaying tumor recurrence by 15 days (Figure 5E). The high sensitivity and high resolution of SERS‐guided surgical resection could potentially be used for the resection of tumors in different organs, and combined treatment could effectively ablate residual tumors.

#### 3.1.3 Guided Tumor Resection Using a Handheld Raman Scanner

Surgical resection is a vital step in the cancer treatment process. However, tiny tumors infiltrate the surrounding tumor.^[^ ^145^ ^]^ Therefore, tumor margins usually are unclear and incompletely resected, leaving small lesions that cause tumor recurrence. SERS can identify these tiny tumor lesions with high sensitivity.^[^ ^144^ ^]^

**Figure 6:**
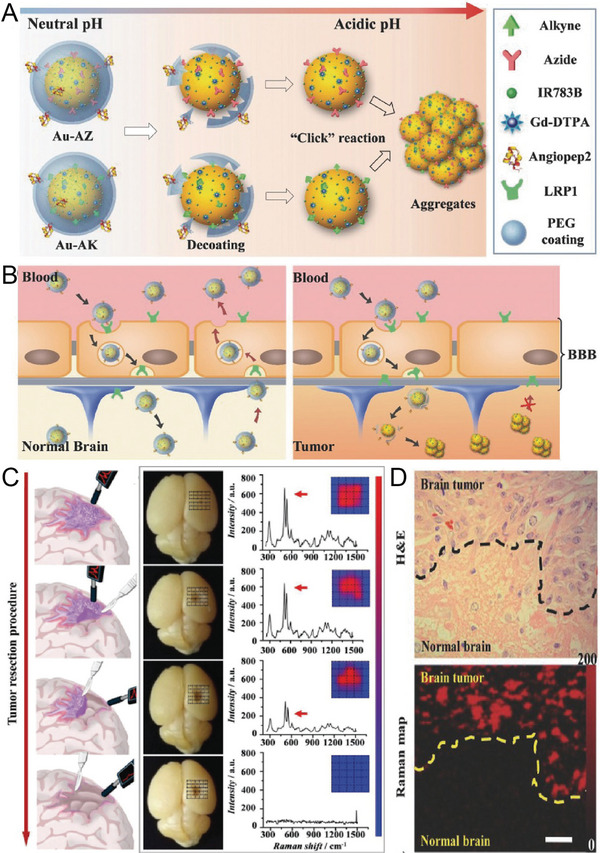
A) Schematic illustration of acid‐responsive Au nanoprobes. The removal of the PEG coating in the tumor environment triggers the aggregation of azide functional AuNPs (Au‐AZ) and alkyne functional AuNPs (Au‐AK) via click cycloaddition reactions. B) The AuNPs are transported back into the bloodstream in normal tissue, while AuNPs can form large aggregates and become trapped in the acidic interstitial environment of the tumor. C) SERS spectroscopy guides the resection of glioma by a handheld Raman detector. D) Hematoxylin and eosin (H&E) staining and Raman mapping of glioma. The dashed line represents the tumor margin. Reproduced with permission.^[^ ^37^ ^]^ Copyright 2017, Wiley‐VCH.

Handheld Raman scanners also play a key role in the recognition of brain tumor margins that cross the blood‐brain barrier (BBB). An important innovation in guiding brain tumor resection was the identification of the extent of microscopic tumors in an engineered RCAS/tv‐a glioblastoma mice model using a handheld Raman scanner in simulated intraoperative situations.^[^ ^121^ ^]^ A previous study reported that transcranial‐focused ultrasound (TcMRgFUS) disrupted the BBB and allowed the transport of 50‐nm or 120‐nm AuNPs to the tumor margin.^[^ ^146^ ^]^ Recently, Li et al. designed a pair of AuNPs to penetrate the brain tumor through the BBB, simultaneously activating magnetic resonance signals (MR) and SERS signals to guide tumor surgery through their specific assembly in acidic tumor environments.^[^ ^37^ ^]^ The nanoprobe was present as a monodisperse NP in a neutral environment. When two probes were intravenously (iv) injected into mice, they entered brain tumors through receptor‐related protein‐1 (LRP1)‐mediated receptor‐mediated transcytosis (RMT) by crossing the BBB. The aggregation of nanoprobes was triggered by a click‐loop addition in the presence of an acidic tumor environment (**Figure** **6**A), thereby activating MR and SERS signals. Intact nanoprobes were transported back to the bloodstream in normal brain tissue, whereas larger aggregates continuously immersed themselves in the tumor stroma, enhancing the specific MR and SERS signals at the tumor site (Figure 6B). MRI was used to study in situ glioblastoma xenografts before surgery, and SERS‐guided tumor resection was realized under a handheld Raman scanner. Enhanced SERS signals were recorded in the tumor region (Figure 6C). SERS signals were almost undetectable in adjacent normal brain tissue after tumor resection, indicating the implementation of intraoperative guided tumor resection with the assistance of a surgical SERS scanner (Figure 6D). Handheld Raman scanners are now in clinical trials, and SERS imaging‐guided surgical procedures are expected to be used for clinical translation.

#### 3.1.4 Accurate Detection and Removal of Intraoperative Residual Microscopic Lesions

**Figure 7:**
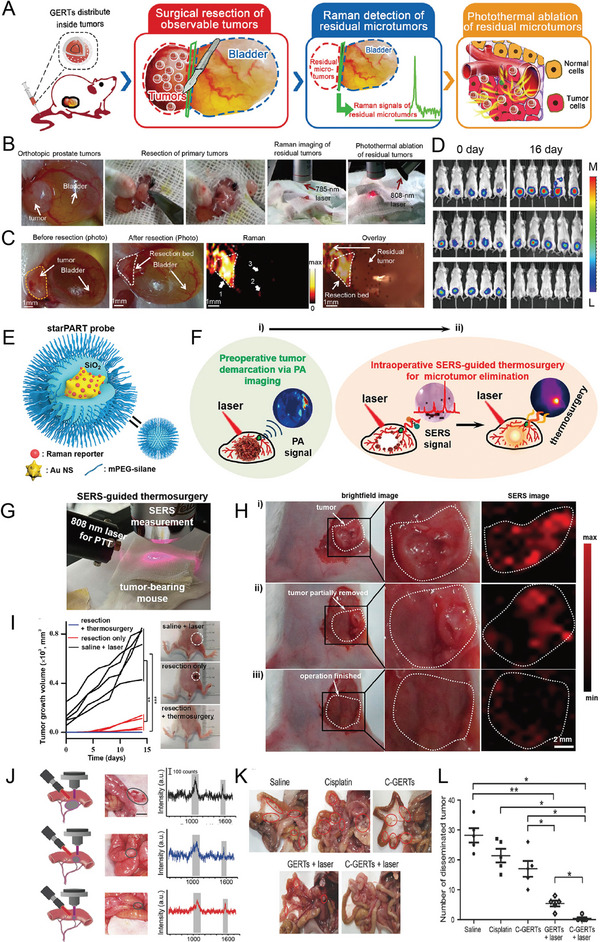
A) Schematic diagram of GERTs for detecting and eradicating the residual tumor. B) Experimental procedure for the PT ablation of residual microtumors guided by Raman imaging. C) Raman imaging for detecting residual microtumors after resecting primary tumors. The yellow dashed line shows the primary tumor, and the white dotted line presents the resection bed. D) Multiple residual tiny tumors (arrow 1), metastasis to the bladder (arrow 2), and normal tissue signal (arrow 3). Reproduced with permission.^[^ ^86^ ^]^ Copyright 2018, American Chemical Society. E) Schematic illustration of the SERS nanoprobe. F) Schematic diagram of PA images for preoperative tumor demarcation and primary tumor resection guidance, and SERS examination of residual tumors and the removal of microtumors. G) Photograph of the experimental procedures. SERS imaging was used for guiding tumor resection and detecting residual microtumors after surgery. H) Brightfield and SERS images i) at the beginning, ii) during, and iii) end of surgery. I) Tumor volume curve of mice with different treatments. Photographs display the tumor outline of mice in different groups. Reproduced with permission.^[^ ^55b^ ^]^ Copyright 2021, American Chemical Society. J) Intraoperative Raman spectra‐guided detection of micro‐tumors and local chemo‐PT therapy of ovarian cancer. K) The treatment effect on ovarian tumors. The red circle represents disseminated tumors. L) The number of disseminated tumors per mouse in each group. Reproduced with permission.^[^ ^68^ ^]^ Copyright 2018, Wiley‐VCH.

The accurate detection and complete removal of tiny tumor lesions is the new strategic goal to reduce local tumor recurrence and treat various tumors.^[^ ^30b^ ^]^ SERS imaging can accurately identify tumor margins and guide the resection of tumor lesions.^[^ ^145^ ^]^ Most noble metal‐based SERS nanoprobes have high PT conversion efficiency. Therefore, they are used in diagnostic platforms in combination with PT therapy. Recently, a SERS nanoprobe was used to accurately detect and remove residual microscopic lesions during intraoperative resection. Xiao et al. applied GERTs to generate sensitive and light‐stable SERS signals for micro‐tumor detection using 785 and 808‐nm high‐power lasers to generate micro‐heat for the ablation of residual microscopic lesions (**Figure** **7**A).^[^ ^86^ ^]^ The results indicated that GERTs identified microscopic tumor lesions with high specificity, also facilitating the removal of microsatellite metastasis (Figure 7B,C), thus achieving accurate tumor resection (Figure 7D).

Li et al. developed “three‐in‐one” therapeutic and diagnostic nanoprobes, such as AuNSts‐based PA, SERS, and thermal surgery (starPART) for PA imaging‐guided tumor surgical resection and subsequent intraoperative SERS imaging to remove residual tiny tumors in thermosurgery.^[^ ^55b^ ^]^ This starPART nanoprobe consisted of an Au nanostar core, a Raman molecular layer, and an outer silica shell (Figure 7E), thus combining the advantages of PA imaging in preoperative deep tissue penetration and SERS detection during intraoperative resection with ultra‐high sensitivity and PT tumor ablation (Figure 7F). The synergistic combination of PA image‐guided tumor resection and SERS‐guided micro‐tumor thermal surgery eradicated the targeted tumors (Figure 7G). SERS imaging of the starPART nanoprobe accurately delineated the tumor edge and guided the micro‐tumor thermal surgery (Figure 7H), eradicating the micro‐tumors with no local recurrence (Figure 7I).

Cisplatin‐loaded gap‐enhanced SERS tags (C‐GERTs) exhibited unique Raman signals when combined with the chemo‐PT synergistic treatment of ovarian cancer, and the removal of microscopic proliferating lesions has been reported (Figure 7J).^[^ ^68^ ^]^ The multifunctional C‐GERTs nanoprobe identified tumors smaller than 0.1 cm while killing tiny tumors that were too close or too small to blood vessels to be removed (Figure 7K,L). This technology allows for the accurate detection and removal of tiny tumor lesions and will be further studied and applied to aggressive tumors and multifocal tumors that are difficult to eradicate.

### 3.2 SERS Sensing of the Physiological Environment

Minor changes in pathological and physiological processes in the organism, including the reactive oxygen species (ROS) content,^[^ ^147^ ^]^ reactive nitrogen species (RNS) content, pH, and corresponding enzymes,^[^ ^148^ ^]^ affect in vivo physiological and pathological states, potentially causing several diseases. Thus, the identification of specific biomarkers is very significant for the early detection of diseases. However, the concentration of biomarkers is relatively low in the early stage of the disease. Thus, the accurate and sensitive detection of changes in the microenvironment of the body allows researchers to understand and study the development of diseases.^[^ ^149^ ^]^ Fortunately, SERS has made rapid progress in the detection of biomarkers in blood, tissues, and tumors, as well as the detection of the tumor microenvironment.^[^ ^150^ ^]^ In this section, the SERS‐based sensing of the living microenvironment and biomarkers is described.

#### 3.2.1 SERS‐Based Sensing of pH in the Tumor Microenvironment

pH,^[^ ^29^, ^151^ ^]^ ROS, RNS, antioxidant molecules, and the corresponding enzymes are involved in cell differentiation, division, apoptosis, and necrosis related to pathological and physiological processes.^[^ ^152^ ^]^ Changes in these components can indirectly indicate the physiological or pathological state.^[^ ^153^ ^]^ Therefore, various biosensor‐based physiological microenvironments have been designed and studied in recent years.

SERS‐based sensing shows a sensitive and specific response to a specific environment and the intensity changes when the local physiological microenvironment changes. A change in SERS intensity is used for diagnosing and monitoring diseases. pH is a vital regulating factor in the physiological environment, affecting ion regulation in nerves and glial cells, and is closely related to chronic degenerative diseases, such as ischemia and Alzheimer's disease.^[^ ^154^ ^]^ Recently, Li et al. successfully developed two pH‐responsive nanoprobes. Acidic tumor conditions trigger the assembly of NPs, forming 3D spherical nanoclusters with significantly enhanced MR and SERS signals.^[^ ^37^ ^]^ This nanoprobe with enhanced SERS accurately identified tumor edges and facilitated the clinical application of AuNS‐based imaging nanoprobes. pH and GSH dual‐responsive nano‐assemblies were also used to distinguish normal tissues from tumor regions.^[^ ^155^ ^]^

**Figure 8:**
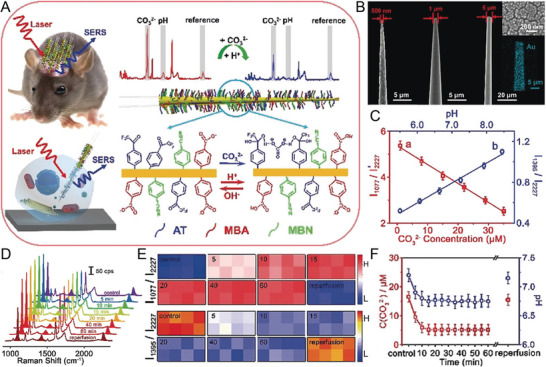
A) The design of a ratiometric SERS probe allows it to simultaneously respond to CO_3_^2−^ and pH in living brains and single neurons. B) SEM images of different tip quartz tapers (QTs) coated with rough gold film. C) Linear relationship between ratiometric SERS intensity and the concentration of CO_3_^2−^ and pH. D) SERS spectra at different time points in the cortex of a live mouse brain upon middle cerebral artery occlusion (MCAO). E) Corresponding ratiometric SERS mapping of I_1077_/I_2227_ and I_1395_/I_2227_. F) The corresponding concentration of CO_3_^2−^ and pH value at different time points in the cortex of a live mouse brain upon MCAO. Reproduced with permission.^[^ ^39^ ^]^ Copyright 2019, Wiley‐VCH.

Carbonate (CO_3_^2−^) is a weak acid that plays a vital role in maintaining homeostasis and adjusting the acid‐base balance. Recently, Tian et al. developed a SERS optical physiological probe for the mapping and accurate detection of pH and CO_3_^2‐^ concentrations in the live brain (**Figure** **8**A).^[^ ^39^ ^]^ It was composed of eight microprobes with a tip size of 5 µm (Figure 8B), and it sensitively, specifically, accurately, and quantitatively monitored pH and CO_3_^2−^. The Raman molecular signals of 4‐mercaptobenzoic acid (MBA) and 1‐(4‐aminophenyl)‐2,2,2‐trifluoroethanone (AT) were used to specifically identify pH and CO_3_^2−^, respectively, and 4‐mercaptobenzonitrile (MBN) was used as inner reference. AT reacted with CO_3_^2−^ via hydrogen bonding with an increased CO_3_^2−^ concentration conditions, resulting in a decrease in SERS intensity at 1077 cm^−1^ from the stretching vibration of the benzene ring of AT. With increasing pH, the COOH group of MBA was converted to ‐COO^−^, and the SERS intensity at 1395 cm^−1^ increased due to COO^−^ stretching. The SERS intensity remained unchanged at 2227 cm^−1^ from 4‐MBN in the ratiometric nanoprobe. With increasing CO_3_^2−^ concentrations and decreasing pH, the ratiometric SERS signal of I_1077_ I_2227_ and I_13952_/ /I_2227_ decreased, demonstrating an excellent linear relationship (Figure 8C). The ratiometric SERS signal of I_1077_ I_2227_ gradually increased and that of I_1395_/ /I_2227_ gradually decreased in the cortex upon MCAO within 10 min and remained stable for 1 h (Figure 8D,E). The concentration of CO_3_^2−^ decreased from 16.50 ± 1.95 to 5.14 ± 2.05 µm, and the pH decreased from 7.20 ± 0.13 to 6.75 ± 0.11 within 10 min (Figure 8F). Thus, this SERS probe successfully realized the simultaneous quantification and detection of chemical species in vivo.

**Figure 9:**
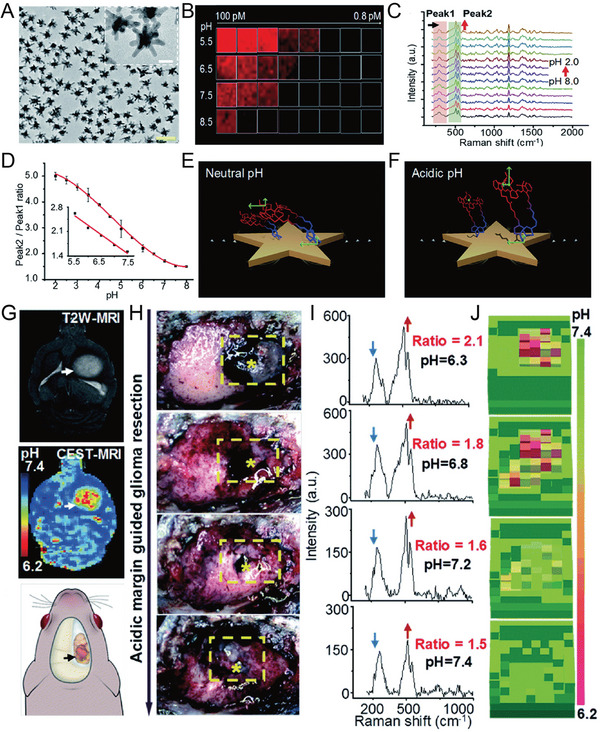
A) TEM images of AuS‐IR7p nanoprobe, scale bar: 100 nm. B) SERS image of AuS‐IR7p at 510 cm^−1^ at different concentrations (0.8–100 pm) and pH values (5.5, 6.5, 7.5, and 8.5). C) Raman spectra AuS‐IR7p at different pH values (each interval is 0.5 units). D) Ratiometric SERS signal decreased as a function of pH. E,F) A protonated IR7p exhibits a parallel conformation for the metal surface, which presents a slant or vertical conformation under acidic conditions. G) Acid‐responsive ratiometric nanoprobe‐guided brain tumor resection of live rat models. Preoperative T_2_‐MR image of an orthotopic rat brain tumor. H) The tissues were excised sequentially until no AuS‐IR7 SERS signals were detected in the operative bed. The yellow dashed boxes mark the region where the SERS signal was detected, and the star symbols indicate the points with a higher ratio. I) Corresponding Raman spectra and J) pH mapping. Reproduced with permission.^[^ ^151a^ ^]^ Copyright 2020, The Royal Society of Chemistry.

The metabolic conversion from oxidative phosphorylation to aerobic glycolysis in tumor cells leads to the acidification of the tumor microenvironment (pH 6.2–6.9).^[^ ^156^ ^]^ Gillis et al. reported that the most infiltrated area around the tumor corresponded to the lowest extracellular pH (pHe).^[^ ^157^ ^]^ Therefore, the intraoperative determination of the acidity of the tumor resection margin is promising for locating and removing the highly malignant infiltration area. Li et al. developed a class of ratiometric pH‐responsive SERS nanoprobes to sensitively, reversibly, and independently measure the pH of the acidic “metabolic boundary” of the tumor, improving the resection rate of glioma and the survival time of the experimental animal model.^[^ ^151a^ ^]^ The pH‐responsive SERS nanoprobe used AuNSt as the substrate to modify the heptamethyne cyanine derivative IR7 or an acid‐responsive Raman molecule IR7p (**Figure** **9**A). The in vitro Raman imaging revealed that the acidic environment triggered the enhancement of the AuS‐IR7p SERS signal, and a low LOD of 0.8 pm was recorded at pH 5.5 (Figure 9B). When the pH was lowered from 8.0 to 2.0, the Raman signal intensities at 527 and 558 cm^−1^ from the ring deformation of the methylene carbon chain showed an elevated trend (Figure 9C). The peak at 311 cm^−1^ from Au—S stretching and bending remained stable, and the intensity ratio of the Raman peak 2 (450–595 cm^−1^) to 1 (280–370 cm^−1^) increased from 1.5 to 5.0, showing a linear relationship in a pH range of 5.5–7.5 (Figure 9D). The protonation of IR7p increased the electrostatic repulsion between the molecules and triggered their orientation from horizontal to oblique or perpendicular to the metal surface, strengthening the vibrating bond in the framework in the EM field compared to the Au—S bond conjugated to the metal surface (Figure 9E,F). The average pH in the highly aggressive in situ C6 glioblastoma in living rats was 6.6, compared to 7.2 in a healthy brain, and a craniotomy was performed in the right frontal‐parietal area (Figure 9G,H). The pH was measured in real‐time under the SERS nanoprobe to evaluate the margins of the malignant tumor in a live animal model, and tumor tissue was gradually removed until no lesions with a pH lower than 7.0 were detected (Figure 9I,J). This strategy significantly prolonged the survival rate of the animal model. Thus, ratio‐responsive SERS nanoprobe‐guided surgery presents great promise in identifying the boundaries of aggressive gliomas and achieving a balance between minimal functional damage and maximum tumor reduction.

#### 3.2.2 SERS‐Based Sensing of ROS and Redox Potential in Diseases

The ROS content is a vital physiological indicator of the tumor microenvironment, referring to the reactive oxyradical produced by oxygen metabolisms,^[^ ^158^ ^]^ such as H_2_O_2_, ^·^OH, ROO^·^, singlet oxygen, and hypochlorous acid (ClO^−^).^[^ ^159^ ^]^ An unbalanced distribution of ROS in the body can change the normal redox balance, causing chronic inflammation and cancer. Therefore, the development of SERS nanoprobes that can effectively explore and detect changes in ROS in physiological and pathological processes is beneficial to the early diagnosis and prevention of different diseases, including cancer. However, the ROS content in the body is low and short‐lived, causing challenges in sensitive, direct, and accurate detection in vivo. SERS‐based ROS sensing has significant advantages at low‐concentration, and low‐intensity signals demonstrate the narrow Raman characteristic signal and are easy to distinguish from background signals.

**Figure 10:**
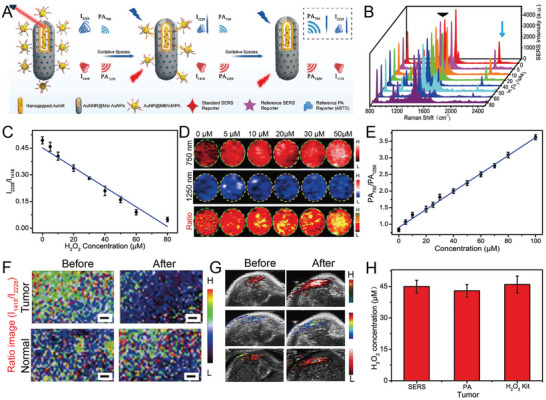
A) The design of an H_2_O_2_‐responsive core‐satellite SERS nanoprobe with ratiometric PA and SERS imaging signals. B) SERS spectra of the nanoprobe incubated with H_2_O_2_. C) The linear relationship of the SERS intensity ratio of I_2228_/I_1418_ and H_2_O_2_ concentrations. D) PA images of a core‐satellite nanoprobe at 750 and 1250 nm and ratiometric images at different H_2_O_2_ concentrations. E) The standard curve of ratiometric PA_750_/PA_1250_ at various H_2_O_2_ concentrations. F) Ratiometric SERS mapping and G) ratiometric PA imaging in tumor‐bearing mice, scale bar: 1 mm. H) The concentration of H_2_O_2_ in the tumor tested by three methods. Reproduced with permission.^[^ ^60c^ ^]^ Copyright 2021, Wiley‐VCH.

SERS imaging has the advantage of high sensitivity. However, its slow imaging speed and shallow penetration depth are often problematic. NIR PA imaging has deep tissue penetration ability and high spatial resolution that efficiently makes up for the disadvantages of SERS imaging. Song et al. developed a H_2_O_2_‐response ratiometric SERS and PA core‐satellite nanoprobes and achieved an accurate and sensitive detection of H_2_O_2_ in tumors and inflammatory sites in mice.^[^ ^60c^ ^]^ The core‐satellite nanostructure (called as AuNNR@MSi‐AuNPs) was composed of amino‐modified mesoporous silica coated with a nanogap Au nanorod core‐shell structure (AuNNR@MSi) and AuNPs modified with 4‐mercaptophenylboronic acid (MPA) and D‐(+)‐galactose prepared by an amidation reaction (**Figure** **10**A). The photoacoustic molecules of horseradish peroxidase (HRP) and 2, 2'azino‐bis (3‐ethylbenzothiazoline‐6‐sulfonic acid) (ABTS) were loaded on the mesoporous silica shell. Borate groups between the AuNPs and the AuNNR@MSi were converted to hydroxyl groups upon reaction with H_2_O_2_, and the AuNPs gradually dissociated. The SERS signal of the 4‐mercaptobenzonitrile (MBN)‐modified AuNPs gradually decreased (Figure 10B), whereas that of 2‐naphthalenethiol (NAT) in the nanogap remained unchanged (Figure 10C). In addition, the ABTS loaded in the mesoporous silica shell was converted to an oxidized state by HRP catalysis. Then, the PA signal gradually increased while the intensity of AuNNRs remained almost unchanged. Therefore, the nanoprobe showed a decrease in the SERS ratio (I_1418_/I_2228_), an increase in the PA ratio (PA_750_/PA_1250_), and displayed a good linear relationship with H_2_O_2_ (Figure 10D,E). The SERS ratio (I_1418_/I_2228_) in the tumor‐bearing mouse model gradually decreased, and the PA ratio (PA_750_/PA_1250_) gradually increased when the H_2_O_2_ concentration increased at the tumor site, thereby achieving the real‐time imaging of the tumor and the quantitative detection of H_2_O_2_ (Figure 10F,G). Commercial H_2_O_2_ detection kits confirmed the accuracy of the results (Figure 10H). AuNNR@MSi‐AuNP nanoprobes have also been successfully applied to acute peritonitis and dermatitis imaging in mice, knee osteoarthritis in rabbits, and the quantitative analysis of H_2_O_2_. When an anti‐inflammatory drug (aspirin) was loaded on the mesoporous silica shell, the nanoprobe successfully realized real‐time imaging and tracking of the therapeutic effect on peritonitis. This strategy provides an efficient method for the in vivo quantitative detection of diseases and the integration of diagnosis and treatment.

The redox state is related to inflammation in joints, and a reduction indicates the initial and secondary damage associated with diabetes.^[^ ^160^ ^]^ In recent work, multiple SERS‐active microneedles were developed to simultaneously detect the redox potential and pH in rat joints.^[^ ^29^ ^]^ Two grooves in the SERS‐active microneedle were equipped with redox‐sensitive and pH‐sensitive SERS probes. These probes not only detected the redox state and the dynamic evolution of pH in muscles with less invasiveness but also allowed for the study of the pH and redox state in rat arthritis. These multiple SERS‐active microneedles offer potential applications in detecting tissue lacking flowable fluids.

#### 3.2.3 SERS‐Based Sensing of Metal Ions

Cu is an important trace metal element in the entire body. Cu ions are the main components of superoxide dismutase and play an important role in preventing oxidative stress in organisms.^[^ ^149^ ^]^ Cu^+^ and Cu^2+^ imbalance is accompanied by an increase in ROS production, causing diseases such as liver damage, kidney damage, Wilson's disease (WD), and Alzheimer's disease. Real‐time monitoring of Cu^2+^ concentrations is of significance for understanding the pathological and physiological processes of oxidative stress damage in diseases.

**Figure 11:**
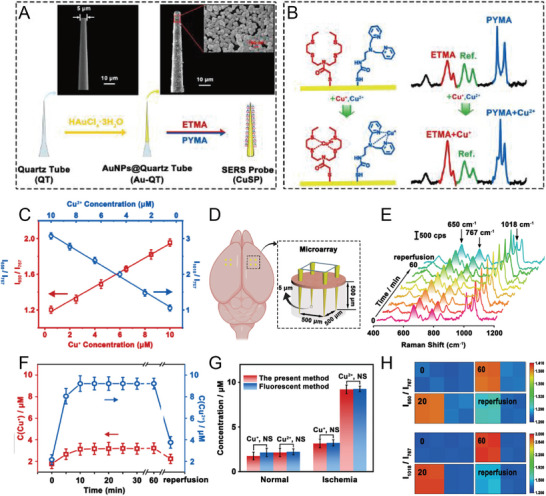
A) SEM images of quartz nanotubes (QTs) with 5‐µm tip sizes and QT coated with AuNPs (Au‐QTs). B) The principle of the SERS nanoprobe identifies Cu^+^ and Cu^2+^. C) Linear relationship between SERS intensity ratio (I_1018_/I_767_ and I_650_/I_767_) and different concentrations of Cu^+^ and Cu^2+^. D) Schematic illustration of SERS microarray for imaging the cortex of mice brains. E) SERS spectra and F) corresponding changes in Cu^+^ and Cu^2+^ concentrations in the cerebral cortex of mice at different time points. G) Concentrations of Cu^+^ and Cu^2+^ tested by SERS or the fluorescence method. H) Ratiometric SERS mapping of I_650_/I_767_ and I_1018_/I_767_ in mice cerebral cortex upon MCAO and reperfusion. Reproduced with permission.^[^ ^123^ ^]^ Copyright 2021, Wiley‐VCH.

However, methods for tracking and quantifying Cu^2+^ and Cu^+^ concentrations in diseases have always been a limitation in studying the CM and pathological processes of neurodegenerative diseases. Tian et al. developed a new SERS probe to monitor and quantify Cu^+^ and Cu^2+^ concentrations in the cerebral cortex of living animals.^[^ ^123^ ^]^ Two organic molecules N, N‐bis(2‐((2‐(ethylthio)ethyl)thio)ethyl)‐2 mercaptoacetamide (ETMA) and N‐(2‐(bis) (pyridin‐2‐ylmethyl)amino)ethyl)‐2‐mercaptoacetamide (PYMA) were synthesized and self‐assembled on a quartz tube with AuNPs deposited on the tip (Au‐QT) (**Figure** **11**A,B). Au‐QT (expressed as CuSP) specifically recognized Cu^+^ and Cu^2+^. A SERS signal at 767 cm^−1^ was applied as a reference to avoid effects from the environment, probe concentration, and light source (Figure 11C). SERS microarray composed of eight CuSP probes was implanted in vivo in the cerebral cortex of mice for tracking and biosensing Cu^+^ and Cu^2+^ after middle cerebral artery occlusion (MCAO) (Figure 11D). The concentration of Cu^2+^ increased by 4.26 times in ischemia, whereas the concentration of Cu^+^ only increased by 1.80 times (Figure 11E–H). Three possible reasons for the increase in Cu^+^ and Cu^2+^ concentrations during ischemia are: 1) Cu^+^ and Cu^2+^ were exported from the neurons upon MCAO, 2) both ions were released from damaged Cu‐containing protein due to the generation of ROS and the decrease in pH during ischemia, and 3) Cu^+^ was transformed to Cu^2+^ with the production of ROS during ischemia. This study presented a way to simultaneously detect Cu^+^ and Cu^2+^ in the living brain and investigate the physiological and pathological mechanisms of oxidative stress and other events, as well as the prevention and treatment of neurodegenerative diseases.

Song et al. developed Cu^2+^‐activated nanogapped gold NPs (AuNNPs) and poly(N‐isopropylacrylamide) (PNIPAM) to monitor the Cu^2+^ content in liver and urine in patients with WD by PA imaging and the ratio SERS, respectively.^[^ ^161^ ^]^ The Cu^2+^‐chelating agent PNIPAM was modified on the shell surface of the AuNNPs. The amino group of PNIPAM coordinated with Cu^2+^ to generate a Cu**—**N bond, resulting in the accumulation of AuNNPs, accompanied by an enhanced plasmonic resonance effect and increases in the SERS signal intensity ratio of I_2223_/I_1378_. The SERS intensity ratio of I_2223/_I_1378_ showed a linear relationship with Cu^2+^ concentrations. The in vitro SERS detection of the clinical urine samples quantitatively detected Cu^2+^ content (≈11.68 mm), which was consistent with the standard clinical data.

#### 3.2.4 SERS‐Based Sensing of Glucose

One of the important applications of SERS biosensing is glucose detection.^[^ ^1b^ ^]^ Blood glucose levels are measured as a routine indicator in the diagnosis and monitoring of diabetes.^[^ ^162^ ^]^ Diabetes patients usually perform frequent fingertip tests to draw fresh blood and monitor glucose levels. This test is inconvenient and painful.^[^ ^163^ ^]^ Therefore, extensive research is being conducted to develop SERS‐based glucose sensors.^[^ ^1b^ ^]^

**Figure 12:**
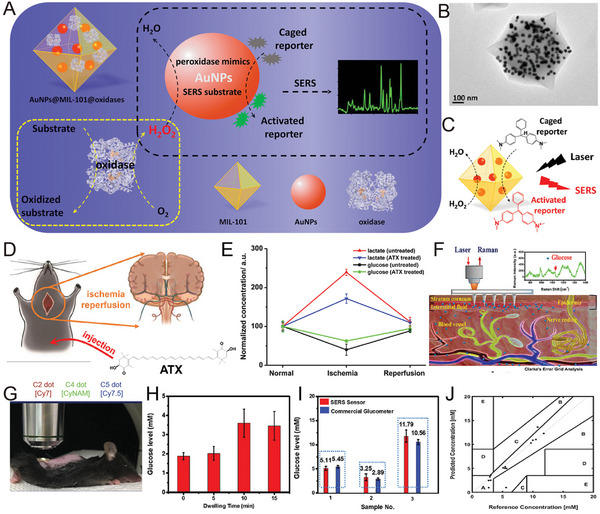
A) Schematic illustration of AuNPs@MIL‐101@oxidase and its catalytic reaction. B) TEM image of AuNPs@MIL‐101. C) AuNPs@MIL‐101, as a peroxidase mimic, catalyzed the conversion of LMG to MG in the presence of H_2_O_2_, which also enhanced its SERS signal. D) Schematic illustration of monitoring glucose, lactate, and ATX treatment in the brain of a live rat with global cerebral ischemia/reperfusion. E) Changes in glucose and lactate levels in the ischemic‐reperfusion brain of mice treated with or without ATX. Reproduced with permission.^[^ ^124^ ^]^ Copyright 2017, American Chemical Society. F) Schematic illustration of measuring glucose levels in vivo using an F‐PMMA MN array based on SERS. G) F‐PMMA MN array applied to scan the skin of a mouse. H) Glucose levels measured using the SERS biosensor. I) Comparison of glucose levels obtained by the SERS glucose biosensor (red) and a commercial blood glucose meter (blue). J) Clark Error grid analysis of in vivo glucose levels obtained in a streptozotocin (STZ)‐induced mouse model of type I diabetes using the SERS glucose biosensor. Reproduced with permission.^[^ ^164^ ^]^ Copyright 2020, American Chemical Society.

Wei et al. reported a nanoprobe by generating AuNPs in situ in a thermally stable and porous metal‐organic framework (AuNPs@MIL‐101) and used them as peroxidase mimics and SERS‐enhanced substrates (**Figure** **12**A).^[^ ^124^ ^]^ AuNPs@MIL‐101 could catalyze the oxidation of cage‐like LMG to Raman active MG and was also used to enhance SERS signals (Figure 12B). Glucose oxidase (GOx) and lactate oxidase (LOx) were then assembled onto AuNPs@MIL‐101 to form integrated nanoenzymes (AuNPs@MIL‐101@oxidases) for the detection of glucose and lactate (Figure 12C,D). AuNPs@MIL‐101@oxidases was successfully used to monitor changes in glucose and lactate levels associated with ischemic stroke in a living brain (Figure 12E). This work demonstrated the great potential of combining the multiple functions of AuNPs for multifunctional bioassays and provided a method for designing high‐performance nanoenzymes for biomedical applications.

Recently, a novel SERS sensor using a poly(methyl methacrylate) microneedle (PMMA MN) array was reported for direct in situ intradermal glucose detection (Figure 12F).^[^ ^164^ ^]^ The array was coated with AgNPs, and then the glucose‐trapped agent 1‐decanethiol (1‐DT) was incorporated into the surface of the Ag‐plated array. The results demonstrated that the functional PMMA MN could measure glucose in interstitial fluid in an STZ‐induced type I mouse diabetes model within a few minutes, maintaining F‐PMMA MN structural integrity (Figure 12G–I). The Clark error grid analysis of the measurement data showed that 93% of the data points were in the A and B areas (Figure 12J). The MN array was invasive to the skin, but the skin recovered well within 10 min after the measurement without any evident adverse reactions. This approach represents an important step toward a minimally painless, invasive, and low‐cost glucose measurement test without the requirement for drawing blood. With further improvements, this polymer MN array‐based SERS biosensor could soon be used for painless blood glucose monitoring by diabetic patients.

#### 3.2.5 SERS‐Based Sensing of Nucleic Acid Biomarkers

SERS is an efficient method for the specific detection of cancer.^[^ ^165^ ^]^ Since various biomarkers are overexpressed in cancer cells, their detection has attracted great attention.^[^ ^166^ ^]^ The detection of biomarkers in vivo is usually based on the recognition of overexpressed antigens on the surface of cancer cells by molecules modified on SERS tags to obtain Raman signals.^[^ ^152^, ^167^ ^]^ SERS detection is based on protein^[^ ^168^ ^]^ and intracellular nucleic acid^[^ ^169^ ^]^ (e.g., DNA,^[^ ^170^ ^]^ RNA, miRNA,^[^ ^171^ ^]^ methylated DNA, circulating tumor DNA (ctDNAs)) biomarkers,^[^ ^172^ ^]^ to facilitate the early diagnosis and prevention of cancer.^[^ ^173^ ^]^

**Figure 13:**
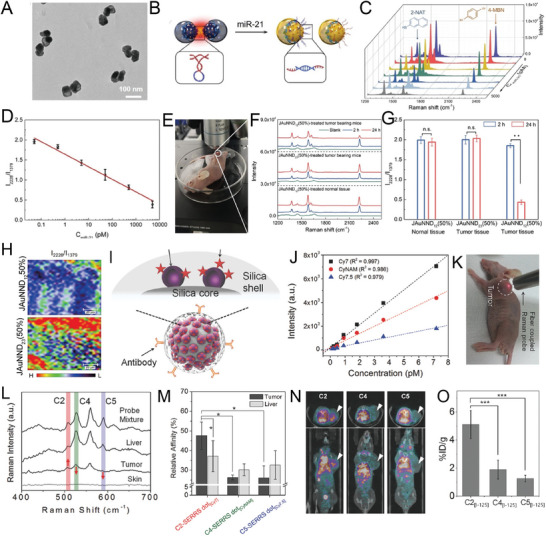
A) TEM image of dimer nanostructure. B) Schematic illustration of triggered disassembly of the dimer into single nanoparticles. C) SERS spectral changes in the dimer nanostructure and D) linear relationship of the SERS intensity ratio (I_2228_/I_1379_) with varying miR‐21 concentrations. E) The tumor site of SERS spectra in a mouse. F) SERS spectra, G) SERS intensity ratio (I_2228_/I_1379_), and H) SERS mapping of tumor site in a mouse treated with a miR‐21‐responsive dimer nanostructure. Reproduced with permission.^[^ ^174^ ^]^ Copyright 2021, Wiley‐VCH. I) Schematic illustration of the SERS nanoprobe. The NIR dye is adsorbed on the Au/Ag hollow‐shell surface, and then a silica nanoshell is coated on its surface. J) Linear relationship between SERS signal intensity and concentrations of the nanoprobe. K) Photograph of fiber‐coupled Raman setup for SERS detection in tumor‐bearing mice. L) SERS spectra from different sites in mice. M) Quantitative analysis of the ratio of the relative affinity of the probes for the tumor and liver. N) CT images of tumor‐bearing mice after the injection of I^125^‐labeled candidate antibodies C2, C3, and C5. The white arrow represents the location of the tumor. O) The in vivo affinity screening of three I^125^‐labeled antibodies at the tumor site. Reproduced with permission.^[^ ^167^ ^]^ Copyright 2018, Wiley‐VCH.

Ratiometric detection helps improve detection accuracy. Recently, a miRNA‐responsive dimeric nanostructure composed of Janus nanogap AuNPs with partial Au shell coverage successfully achieved miRNA imaging and detection at the tumor site in mice (**Figure** **13**A).^[^ ^174^ ^]^ SERS‐based miR‐21 ratiometric detection was developed (Figure 13B–D) using DNA‐driven dimeric nanostructures (“core‐to‐core”) and was successfully used for miR‐21 imaging and detection (Figure 13E–H). The SERS‐based ratiometric nanoprobe was a powerful tool for miRNA detection.^[^ ^175^ ^]^

Antibody‐based therapies are rapidly emerging for the treatment of diseases, especially cancer, and immune diseases. A useful verification method needs to be established to select antibodies with the required functions and verify the candidate antibodies in vivo after in vitro antibody screening. SERS is an excellent multiple‐validation tool for antibody candidates. Lee et al. reported the in vivo validation of anti‐tetraspanin‐8 antibody candidates for colon cancer using the SERS nanoprobe ratiometric quantitative method (Figure 13I).^[^ ^167^ ^]^ About 93% of the NIR‐SERS hotspots were detected at the single‐particle level, and the signal intensity was 100 times stronger than that of spherical AuNPs (80 nm) labeled with non‐resonant molecules (Figure 13J). The pilot validation of antibodies in human colorectal cancer xenografts in mice indicated that multiple quantitative analyses could overcome the key issue of inter‐subject variability (Figure 13K–M) and was comparable to the conventional methods using single‐photon emission computed tomography (CT) (Figure 13N,O). In conclusion, SERS‐based sensors provide a sensitive and reliable method for monitoring changes in the microenvironment under pathological and physiological conditions that are conducive to the early diagnosis and prevention of diseases.

### 3.3 SERS in Combination with Other Imaging Methods as a Diagnostic Tool

SERS‐related multimodal nanoprobes^[^ ^176^ ^]^ have been developed in recent years to make good use of the advantages of SERS probes,^[^ ^177^ ^]^ and include SERS combined with fluorescence imaging,^[^ ^178^ ^]^ PA imaging,^[^ ^179^ ^]^ CT,^[^ ^129^ ^]^ positron emission tomography (PET),^[^ ^180^ ^]^ and MRI.^[^ ^115^ ^]^ Multimodal imaging technology combines the advantages of the respective imaging technologies and is beneficial to improving the sensitivity, specificity, and accuracy of detection, providing multi‐dimensional information on biological processes.

#### 3.3.1 Dual‐Modality Imaging as a Diagnostic Platform

**Figure 14:**
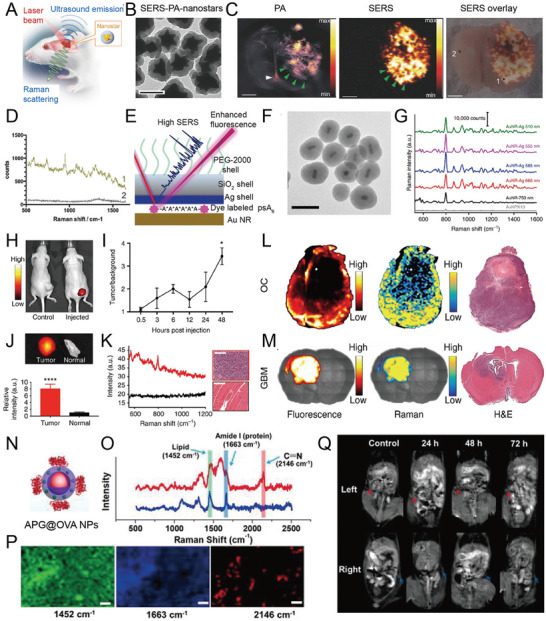
A) Schematic illustration of AuNSts coated with Raman reporter and silica shell for dual‐modality SERS and PA imaging of glioblastoma. B) TEM image of SERS‐PA‐nanostars, scale bar: 100 nm. C) SERS and PA imaging of tumor. D) SERS spectra of tumor (1) and normal tissue (2). Reproduced with permission.^[^ ^125^ ^]^ Copyright 2018, Wiley‐VCH. E) Schematic diagram of the design of a SERS nanoprobe. F) TEM image of AuNRs core with fluorophore masking and a 2‐nm Ag shell and silica shell. G) SERS spectra of AuNP‐based fluorescence‐Raman bimodal NPs (FRNPs) at 10 fm and AuNR‐based FRNPs at 1 fm with increases in Ag thickness. H) Fluorescence images of ovarian tumor‐bearing mice after the injection of 200 µL of 1 µm DylightTM‐780 labeled DNA (left) and 200 µL of 10 nm FRNPs (right) at 48 h. I) The corresponding fluorescence signal at different time points. J,K) The fluorescence signal intensity of the tumor was about 8 times higher than that of normal tissue. L) Fluorescence and SERS images of the resected ovarian cancer tumor with H&E sections of the adjacent tissues. M) High correlation between fluorescence and SERS images of coronal sections with adjacent H&E histology in mice glioblastoma. Reproduced with permission.^[^ ^126^ ^]^ Copyright 2019, Springer Nature. N) Schematic illustration of Au@Prussian blue‐Gd@ovalbumin NPs (APG@OVA NPs). O) SERS spectra of inguinal lymph node treated with or without APG@OVA NPs. P) SERS mapping of inguinal lymph node tissue, scale bar: 20 µm. Q) MR image of tumor‐bearing mice treated with APG@OVA NPs labeled with bone marrow‐derived dendritic cells (BMDCs) at the left lymph node. Reproduced with permission.^[^ ^127^ ^]^ Copyright 2020, Ivyspring International.

SERS has the advantages of high sensitivity and specificity, but its imaging speed is low. The current Raman scanners slowly collect spectral signals point‐by‐point. At least a few minutes are required to image mice, and it is difficult to accurately study the clinically relevant areas in real‐time.^[^ ^129^ ^]^ A dual‐mode SERS‐PA‐nanostar contrast agent was designed to address the above problem and demonstrated a high degree of infiltration of glioblastoma in clinically relevant mouse glioblastoma models by Raman and PA imaging (**Figure** **14**A,B), enabling high signal specificity and sensitivity for brain depths of several millimeters.^[^ ^125^ ^]^ SERS enables unique and sensitive high‐resolution surface inspections, but it is more time‐consuming and has a limited depth (Figure 14C,D).

Recently, multifunctional fluorescence‐SERS bimodal probes combined with fast fluorescence imaging and SERS technology were designed for better in vivo imaging.^[^ ^179^ ^]^ Recent research used DNA as a programmable link between AuNP and fluorophores to design FRNPs for FI and SERS imaging, combining the high speed of FI with the high sensitivity of SERS (Figure 14E–G).^[^ ^126^ ^]^ FRNPs selectively accumulated in the tumor site, real‐time tumor detection was performed by FI (Figure 14H–J), and Raman spectroscopy was used to identify the tumor edge of the resected tissue (Figure 14K–M). Thus, the design of such FRNPs may provide a potential tool for clinical imaging and the detection of cancer by the combination of fluorescence and Raman‐based approaches.

The monitoring of the migration of dendritic cells (DCs) to the lymphatic system is vital in evaluating immunotherapy. MRI exerts no radiation damage, has excellent tissue imaging ability, and combined with SERS imaging, can provide information about DC migration and colonization. Liu et al. developed a bimodal Prussian blue (PB)‐coated AuNP imaging agent called Au@Prussian blue‐Gd@ovalbumin NPs (Au@PB‐Gd@OVA NPs) for in vivo DC activation and the monitoring of DC migration in real‐time by SERS and MRI. PB was assembled on the AuNPs as a cyanide (CN)‐bridged coordination polymer, providing a strong and background‐free SERS signal (Figure 14N).^[^ ^127^ ^]^ The doped Gd^3+^ in the PB lattice demonstrated highly sensitive MR imaging ability without affecting Raman intensity. Au@PB‐Gd@OVA NPs activated and labeled DCs due to the presence of ovalbumin (OVA) and also monitored the migration of DCs in vivo and accurately analyzed their distribution in the lymphatic system by SERS/MR bimodal imaging (Figure 14O,P). MR imaging showed that APG@OVA NP‐labeled DCs migrated from the footpad to the sentinel lymph node in a time‐dependent manner. Raman mapping of lymph node tissue sections revealed that the activated DCs were successfully implanted into the sentinel lymph nodes (Figure 14Q). These experiments revealed that APG@OVA NPs had high activation efficiency and dual complementary imaging performance and could be used as a high‐performance tracer for DC‐based immunotherapy.

#### 3.3.2 Multimodal Imaging for Diseases Detection

**Figure 15:**
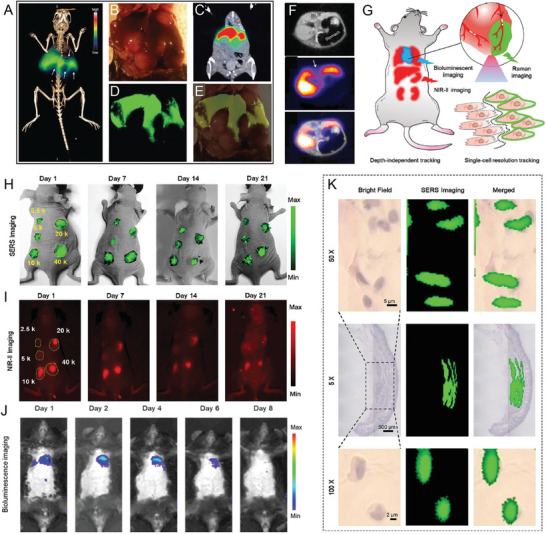
A) Preoperative PET imaging of the whole body of mice. B) Intraoperative white light image of the liver. C) PET imaging of a healthy liver (high signal). D) Intraoperative SERS imaging shows the location and extent of the tumor. E) Overlay of white light image and SERS mapping. F) PET‐MR images show significant filling defects (arrows) in the liver. Reproduced with permission.^[^ ^130^ ^]^ Copyright 2017, Ivyspring International. G) Schematic illustration of Au nanostar‐3.3′‐diethylthiatricarbocyanine iodide‐silver sulfide NPs (termed Au‐Star‐DTTC‐Ag2S NPs, GDC) for tracking therapeutic mesenchymal stem cells (MSCs) in myocardial infarction (MI). The survival of MSCs in the heart tissue is illustrated by BLI, and the distribution and metabolism in vivo are demonstrated by NIR‐II fluorescence imaging. H,I) In vivo SERS/NIR‐II fluorescence imaging in hypodermic models after injection with different numbers of GDS‐MSCs (2.5, 5, 10, 20, and 40 K cells). J) BLI of MI in mice. K) SERS mapping of single cells at different magnifications after injection with GDS‐MSCs. Reproduced with permission.^[^ ^181^ ^]^ Copyright 2021, Wiley‐VCH.

SERS imaging can effectively observe tiny cancerous tumor tissue. However, its slow speed limits the number of cancerous tissues observed during surgery, and it is difficult to realize preoperative surgical imaging. Gambhir's team reported the first trimodal NPs (i.e., MPR NPs) combined with MRI, PA, and Raman imaging to detect mouse brain tumor margins.^[^ ^128^ ^]^ Recently, a new method was developed using radiolabeled SERS NPs and PET‐SERS tumor imaging without using adhesion molecular chelators.^[^ ^104^ ^]^ PET is used to locate the macroscopic distribution of the tumor inside the organ using suitable silicon‐encapsulated chelator‐free radiolabeling before surgery, and then the tumor is confirmed using a Raman imaging device or a handheld Raman scanner. Preoperative whole‐body imaging signals showed several different filling defects in the liver (**Figure** **15**A), while SERS detection exposed the liver of the mouse, indicating some large tumors at the exact position, which correlated with the size and location of filling defects (Figure 15B–F). The true degree of diffusion increased imaging accuracy, providing high‐precision guidance during the procedure.

In the past two decades, stem cell‐based therapy has been used as an effective method for various heart disease treatments. It is very important to trace the migration, metabolism, distribution, and other cellular behaviors of transplanted stem cells. However, BLI technology can only trace living cells, and its poor imaging accuracy further limits its application in monitoring the behavior of stem cells in vivo. SERS imaging has an extremely narrow specific spectral fingerprint, presenting a novel method for the ultra‐high sensitivity and single‐cell resolution imaging of deep tissue. Zhou et al. developed AuNSt nanoprobe‐based NIR‐II FI/SERS imaging (Au nanostar‐3.3'‐diethylthiocarbocyanine iodide‐Ag sulfide NPs, Au‐Star‐DTTC‐Ag_2_S NPs) for labeling and accurately tracking MI mesenchymal stem cells (Figure 15G).^[^ ^181^ ^]^ Ag_2_S NPs produced NIR‐II emission, which enabled the observation of cell behavior (Figure 15H,I). Subsequently, the SERS signal of the Au‐Star‐DTTC chamber realized high‐resolution Raman imaging (Figure 15J), and stem cells in the surrounding normal tissues were effectively visualized. The boundary between normal heart tissue and the MSCs was depicted according to their SERS signal (Figure 15K). This imaging and tracking method displayed depth‐independent and high‐resolution imaging abilities.

### 3.4 SERS‐Guided Multifunctional Diagnosis and Treatment Platforms

The combination of SERS imaging and therapy can diagnose tumors with high sensitivity and serve as a multifunctional theranostic platform.^[^ ^35^ ^]^ Therefore, the multifunctional therapeutic platform guided by SERS imaging and multimodal imaging and treatment combined with other imaging technologies has received extensive attention in the last few years. Multimodal imaging can greatly improve diagnostic accuracy, showing great potential in preclinical research and translational imaging. PTT based on the properties of the nanomaterial, including targeting, drug delivery, and combined chemotherapy with nano drugs, allows for the treatment of many diseases.^[^ ^101^, ^182^ ^]^ In the next section, the latest developments in SERS combined drug release monitoring and treatment, SERS‐guided theranostics, and multifunctional treatment platforms are reviewed in detail.^[^ ^183^ ^]^

#### 3.4.1 SERS‐Guided Theranostic Platforms

**Figure 16:**
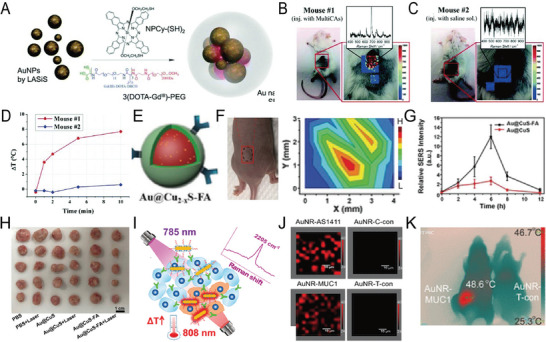
A) Schematic illustration of the synthesis of the MRI/SERS contrast agent (MultiCA). B,C) SERS imaging of regions #1 and #2 in mice. D) Temperature changes in a tumor without irradiation. Reproduced with permission.^[^ ^115^ ^]^ Copyright 2018, The Royal Society of Chemistry. E) Schematic illustration of Au@Cu_2−x_S core‐shell NPs. F) Photograph of tumor‐bearing mice. Corresponding SERS intensity of a black ellipse. G) SERS spectra of a tumor at different time points. H) Photograph of tumor tissues of mice in different groups. Reproduced with permission.^[^ ^45b^ ^]^ Copyright 2019, Wiley‐VCH. I) SERS nanoprobe as a theranostic platform for cancer imaging and therapy. J) SERS mapping of the tumor. K) Temperature images of mice after irradiation with an 808‐nm laser (1 W cm^−2^, 10 min). Reproduced with permission.^[^ ^185^ ^]^ Copyright 2021, American Chemical Society.

SERS‐based theranostic platforms have received extensive attention in biomedical research.^[^ ^46b^ ^]^ Bhatia et al. successfully achieved multiplexed Raman imaging and PT therapy for the first time, using the NIR plasmonic resonance of AuNRs.^[^ ^78^ ^]^ The combination of a SERS nanoprobe with other imaging techniques was reported for multiple diagnoses and treatments of cancer.^[^ ^184^ ^]^ Recently, Meneghetti et al. developed a SERS/MRI multimodal contrast agent based on bare AuNPs modified with Gd(_III_)‐loaded PEG polymers for cancer imaging and local hyperthermia. First, bare AuNPs were synthesized by the laser ablation method, and then naphthalocyanine was functionalized with a 3DOTA‐PEG polymer MRI contrast agent with high Gd(_III_) loading (**Figure** **16**A). A bright SERS signal was recorded in the tumor after the intravenous injection of the nanoprobe owing to its enhanced MRI and SERS imaging contrast (Figure 16B,C). The temperature in the tumor region of mouse #1 increased by 8°C compared to the temperature of the head, and the change in mouse #2 was negligible after irradiation at 785 nm, indicating the enhancement of the PT effect in mice (Figure 16D).^[^ ^56^, ^81^ ^]^

Recently, nanostructured Au@Cu_2_‐xS NPs with high PT conversion efficiency (45%) and enhanced PA and SERS signals were synthesized^[^ ^45b^ ^]^ (Figure 16E). The gradual weakening of SERS signals from the middle to the edge accurately located the edge of the tumor boundary and was a more sensitive and reliable method than other methods, such as MRI (Figure 16F). The concentration of intravenous NPs was also obtained by the overall SERS signal of the entire tumor, and the PA results were consistent with the SERS measurement results (Figure 16G). The combination of Au@CuS‐FA NP‐targeted in vivo PA and SERS imaging accurately located a deep tumor and identified the tumor edge, thereby achieving accurate and effective PPT ablation of the tumor (Figure 16H). Tang et al. synthesized a novel lipoic acid‐conjugated bio‐orthogonal Raman reporter with a linear di‐alkynyl structure and functionalized it on AuNRs with aptamers and a bio‐Raman silenced reporter for cancer diagnosis and treatment.^[^ ^185^ ^]^ The Raman reporter molecule and aptamer oligonucleotide were co‐anchored on the AuNR surface (Figure 16I). This therapeutic and diagnostic platform combined with the excellent PT capabilities of AuNRS can be used for both in vivo bioorthogonal SERS imaging and the PTT of cancer. The SERS spectroscopy detection and imaging of tumors in living mice were successfully realized after the intravenous injection of SERS nanotags (Figure 16J). Further, the aptamer‐conjugated SERS nanotag significantly inhibited tumor growth after irradiation, with an inhibition rate of 99% (Figure 16K). These results showed that SERS nanotags could simultaneously and effectively provide both an accurate cancer diagnosis in vivo and PT therapy.

#### 3.4.2 SERS Combined with Other Technologies as Multifunctional Theranostic Platforms

**Figure 17:**
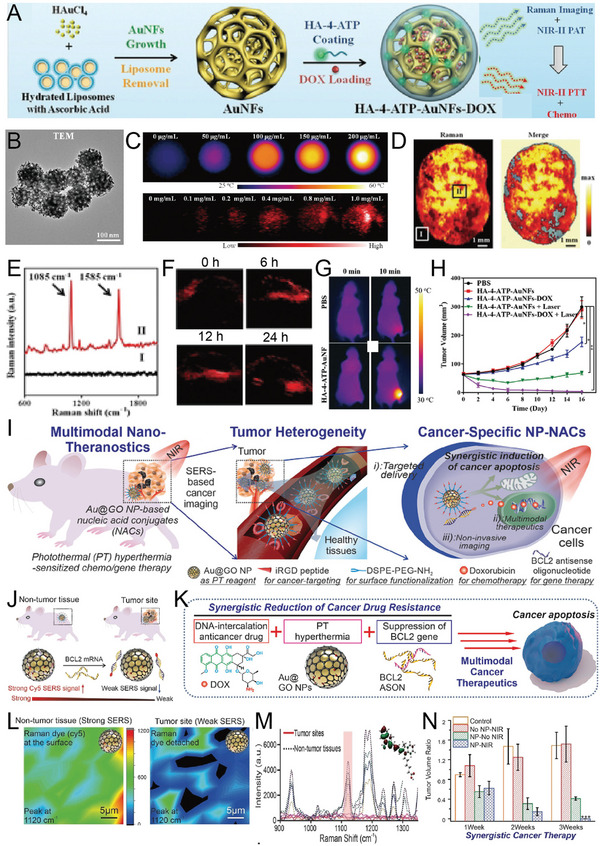
A) Schematic illustration of the synthetic process of a nanoprobe. B) TEM image of gold nanoframeworks (AuNFs). C) PT images and PA images of AuNFs. D) SERS mapping of tumor tissue. E) SERS spectra obtained from positions I and II of the tumor. F) PA images of a tumor at different time points. G) PT images of MDA‐MB‐231 tumor‐bearing mice under 1064‐nm laser irradiation (1 W cm^−2^, 10 min) after injection with phosphate‐buffered saline or an AuNF nanoprobe. H) Growth curves of tumors in different groups. Reproduced with permission.^[^ ^131^ ^]^ Copyright 2020, Wiley‐VCH. I) Schematic illustration of graphene oxide/AuNP‐nucleic acid conjugates (Au@GO NP‐NACs) for in vivo imaging and therapy. J) Schematic illustration of the principle of SERS signal changes. K) Synergistic cancer treatment mechanism of Au@GO NP‐ASON. L) SERS mapping in a tumor and normal tissues. M) In vivo SERS spectra. N) Tumor volume changes in different groups. Reproduced with permission.^[^ ^47a^ ^]^ Copyright 2020, Wiley‐VCH.

Recently, SERS‐guided multimodal imaging theranostic platforms were reported, achieving high‐speed fluorescence imaging, specific SERS imaging, and the PTT ablation of tumors (**Figure** **17**A).^[^ ^126^ ^]^ A study based on a novel mesoporous gold nano framework (AuNF) established a combined photo/thermochemical cancer treatment method, enabling PA/Raman image‐guided tumor photo/thermochemotherapy.^[^ ^131^ ^]^ The novel AuNF with mesopores (≈40 nm) was prepared using a liposome template (Figure 17B) that exhibited a strong NIR‐II PA signal (Figure 17C). Raman molecules on the AuNF surface simultaneously enhanced Raman signals attributed to the high‐density hotspots of the AuNFs (Figure 17D,E). The functionalization of the AuNFs with hyaluronic acid (HA), loaded with DOX in the mesopores, was used to specifically target overexpressed CD44 on tumor cells (MDA‐MB‐231) (Figure 17F). Both in vitro and in vivo (tumor‐bearing xenograft tumor) assessments suggested that HA‐4‐ATP‐AuNFs‐DOX exerted a very high therapeutic effect in tumors by PA‐Raman‐guided photo‐chemotherapy (Figure 17G,H).

Various combinations of in vivo SERS imaging and photodynamic therapy (PDT) techniques have also been performed in mice. He et al. constructed triple‐modal imaging based on SERS‐coded gold nanorods (GNR) by combining PDT with fluorescence imaging.^[^ ^54^ ^]^ They designed multilayer‐coated GNRs that chemically doped Raman and fluorescent dyes in different layers of silica polymer‐modified GNRs for SERS and Raman imaging. A PDT photosensitizer molecule of protoporphyrin IX (PpIX) was carried on the multilayer shell, and PDT was performed after the tumor position was determined. Lee et al. developed multifunctional hybrid rGO oxide/AuNP‐based nucleic acid conjugates (NACs) (Au@GO NP‐NACs) to target nucleic acids in cancer. The material displayed significant changes in Raman signals, exhibiting multimodal synergistic cancer therapy by combining PT, genetic, and chemotherapeutic methods (Figure 17I).^[^ ^47a^ ^]^ SERS imaging of tumors was performed using Raman dye (Cy5)‐labeled nucleic acids (AntiSense OligoNucleotide (ASON) to specifically target and bind cell surface oncogenes (BCL2) (Au@GO NP‐BCL2‐NACs) via *π*‐*π* stacking. More specifically, the delivery of Au@GO NP‐BCL2‐NACs combined with Cy5 Raman dye (Au@GO NP‐Cy5‐BCL2‐NACs) into cancer cells induced a selective binding between ASON and overexpressed BCL2 mRNA, causing the disruption of *π*‐*π* stacking and the subsequent release of Cy5 molecules through a double‐strand formation, thus reducing the SERS signal (Figure 17J). DOX delivery, the suppression of expression of the drug‐resistance gene (BCL2) through antisense ASON, and phototherapy were combined in one platform to verify its therapeutic effect (Figure 17K). In vivo SERS signals at the tumor site were lower than those of the surrounding tissue after one day of injection of the NPs, suggesting that cancer cells exhibited higher BCL2 expression in vivo after the injection of Au@GO NP‐Cy5‐BCL2‐NACs (Figure 17L,M). The tumor was almost completely removed at the end of the in vivo experiment (3 weeks) from the mice treated with PT hyperthermia combined with gene and chemotherapy (Figure 17N), whereas the three control groups did not show any significant changes. The treatment based on Au@GO NP‐NACs demonstrated excellent performance in tumor ablation and had the least toxic effect on non‐cancer cells in vivo, which are requirements for precise cancer treatment. In short, the development of an Au@GO NP‐NAC multifunctional nanotherapeutic platform may pave the way for the next generation of precision medicine, effectively predicting cancer and other diseases complicated by multiple genetic factors and epigenetic changes.

In summary, the diagnostic and treatment platform based on the combination of SERS and other optical imaging and treatment methods showed a wide application potential in future biomedicine. Thus, drug‐carrying, drug release, treatment effects based on SERS probes, and multifunctional theranostic tools combined with multimodal imaging and treatment can not only improve detection accuracy but also facilitate the treatment of diseases.

### 3.5 SERS Progress in Instrumentation and Translation

#### 3.5.1 SERS Progress in In Vivo Imaging Driven by Instrumentation Improvements

SERS‐based targeted detection, multiple imaging abilities, combined multimodal imaging therapy platforms, portable Raman testers, and clinical endoscopes^[^ ^33^ ^]^ offer great possibilities in clinical medicine.^[^ ^187^ ^]^ The design of Raman imaging instruments, endoscopy, and intraoperative imaging for small animals are conducive to promoting the clinical translation of SERS.^[^ ^188^ ^]^ Traditional SERS imaging has a long acquisition time and a small imaging field, limiting the development of in vivo SERS detection and imaging.

A rapid, wide‐area instrument for in vivo small animal Raman imaging (SARI) *was* developed that can image a wide area (> 6 cm^2^) of mice without moving the animals, achieving 10 times faster SERS imaging.^[^ ^189^ ^]^ The SARI system could accurately study four types of signals in the whole trunk (31.25 × 17.5 mm^2^) of a mouse in 30 min and identify SERS signals focusing on a narrower region (18.75 × 12.5 mm^2^) of the liver in 15 min. However, it is still difficult to apply SERS in large animals and humans since SERS needs to work on imaging larger regions in deeper tissues.

**Figure 18:**
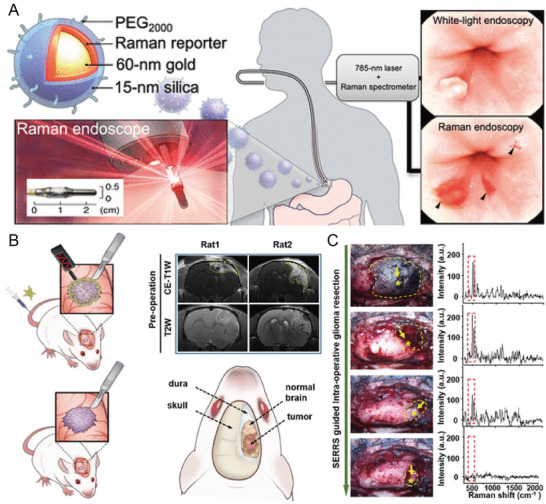
A) Raman endoscopy enabled the detection of tiny lesions of 0.5–1.0 mm, whereas traditional white‐light endoscopy could only detect tumor lesions with a size of 5–7 mm. Reproduced with permission.^[^ ^38b^ ^]^ Copyright 2019, American Chemical Society. B) Illustration of SERS imaging‐guided glioma surgery. Preoperative MR images of the brain in a glioma‐bearing rat. C) Photographs of SERS‐guided glioma resection of a rat bearing an orthotopic glioblastoma xenograft. Reproduced with permission.^[^ ^186^ ^]^ Copyright 2019, American Chemical Society.

Recently, combined SERS and endoscopy have been extensively studied in the detection of disease.^[^ ^33^, ^188^ ^]^ The combination of clinical endoscopic imaging with SERS NPs allows real‐time detection, avoids the systemic toxicity of SERS NPs, and more sensitively detects lesions.^[^ ^190^ ^]^ In addition, the Raman endoscope has higher sensitivity and more accurate detection compared to the current white‐light endoscopy. The lesions detected by white light endoscopy are at least 5–7 mm in size, whereas Raman endoscopy detects lesions of 0.5–1.0 mm. Kircher et al. used PEGylated SERS NPs as probes (**Figure** **18**A) and a customized small animal Raman endoscope and clinically applied them to detect early gastrointestinal (GI) cancer.^[^ ^38b^ ^]^ The results demonstrated that SERS NPs could detect tiny precancerous lesions in real‐time. The Raman endoscope was also applied in real‐time to sensitively detect tumors in a rat model. The real‐time dual‐modal SERS endoscopes have also been used in humans for clinical validation.^[^ ^33^ ^]^ The silica shell generally biodegrades within a few days, leaving the Au core metabolically inert, which is not expected to cause long‐term toxicity. Notably, extensive long‐term toxicity studies are needed before applying the tool to larger animal models and humans. Soon, SERS endoscopy may become an auxiliary tool for routine white light endoscopy to guide the identification of precancerous lesions in the gastrointestinal tract, ultimately achieving the early prevention of cancer.

Spectroscopic pens are used as handheld devices and NIR contrast agents to detect malignant tumors.^[^ ^133^ ^]^ Such devices can effectively remove the absorption of silicon‐carbon rods in optical fibers and weaken the reflected excitation light so that fluorescence and Raman signals can be sensitively analyzed.^[^ ^191^ ^]^ Portable handheld Raman detectors are widely used in cancer detection and diagnosis due to their convenient operation, high portability, and fast acquisition rate. Recently, handheld Raman detectors were employed to guide the intraoperative resection of tumors. The guided resection using a handheld Raman detector was more accurate than under white light and could more accurately detect tiny tumors.^[^ ^121^ ^]^ A recent study showed that a handheld Raman scanner could accurately delineate the tumor‐infiltrating edge outside the adjacent healthy tissue by detecting the characteristic SERS peaks of AuNPs functionalized with Raman molecules (Figure 18B).^[^ ^186^ ^]^ The handheld Raman scanner could visualize and completely remove a glioma xenograft and its invasive margin during surgery. SERS imaging found more minor residual lesions compared to enhanced MRI (Figure 18C), and the postoperative evaluation also showed that the recurrence rate of glioma treated by SERS‐guided surgery was lower. The SERS probe can be used under a handheld Raman tester to guide the resection of different invasive tumors in the future and reduce tumor recurrence rates.

The conventional Raman system only penetrates a depth of several millimeters.^[^ ^192^ ^]^ Surface‐enhanced spatial‐offset Raman spectroscopy (SESORS)^[^ ^133^ ^]^ is used to detect SERS NPs via combining SORS^[^ ^193^ ^]^ with SERS.^[^ ^1^, ^194^ ^]^ SERS NPs can be detected at several centimeters of depth.^[^ ^34^, ^194^ ^]^ Stone et al. reconstructed the multiplexed SERS signaling and SERS mapping of 20 mm‐thick tissue according to their characteristic peak intensities. The original spectra showed that SERS was identified even in 45–50 mm‐thick samples.^[^ ^34^ ^]^ A more recent study demonstrated that the SERS spectra of SERS NPs coated with nanotags successfully penetrated bones of different thicknesses (3–8 mm, the average thickness of the human skeleton) through SESORS.^[^ ^195^ ^]^ In another study, SERS NPs were detected in 3D breast cancer multicellular tumor spheroids (MTS) through 15 mm of porcine tissue using handheld surface‐enhanced spatially offset resonance Raman spectroscopy (SESORRS), and a 25‐mm penetration depth was detected in pig tissues.^[^ ^133^ ^]^ Taken together, SESORS significantly improved penetration, thus helping clinical translation.^[^ ^196^ ^]^

#### 3.5.2 Application of SERS in Larger Animals and Clinical Trial

**Figure 19:**
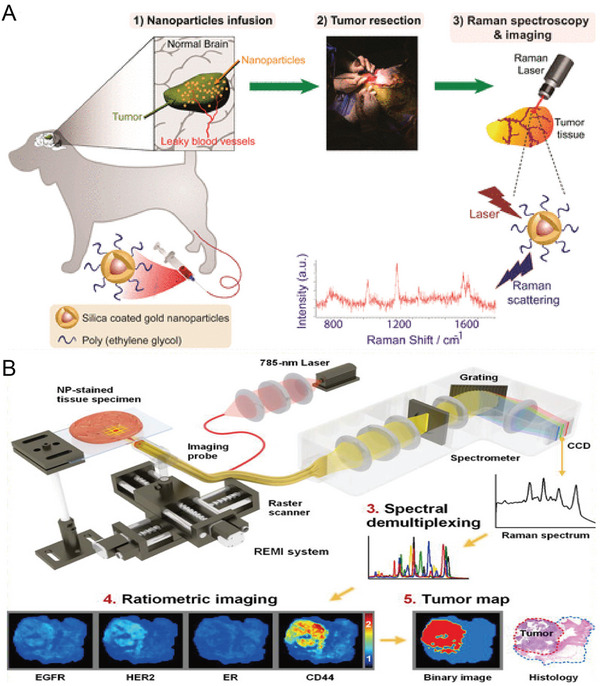
A) Schematic of the overall process of silica‐coated AuNPs for imaging and detecting tumor tissue. Reproduced with permission.^[^ ^38a^ ^]^ Copyright 2019, American Chemical Society). B) Schematic illustration of the overall process of Raman‐encoded molecular imaging (REMI), including staining, imaging, and spectral demultiplexing. Reproduced with permission.^[^ ^135^ ^]^ Copyright 2017, American Association for Cancer Research.

From the clinical detection point of view, SERS‐based multiplex imaging, as a diagnostic platform for multimodal imaging and treatment and guiding tumor resection during surgery, has shown highly promising developments in clinical translation.^[^ ^197^ ^]^ Recently, SERS has been widely used to detect tiny tumor foci and guide tumor resection.^[^ ^45a^ ^]^ Gambhir et al. used spontaneous brain tumors in dogs as a model (very similar to human brain tumors) and applied an Au@SiO_2_ SERS nanoprobe for sensitive and micro‐resolution SERS detection of canine spontaneous brain tumors.^[^ ^38a^ ^]^ The heterogeneous Au@SiO_2_ SERS NPs were observed in different levels of the oligodendroglioma and meningiomas. However, no Au@SiO_2_ SERS was detected in tumor necrosis sites or the normal brain, indicating their EPR effect. The main feature of this experiment was the use of a dog tumor model (**Figure** **19**A), allowing the assessment of NP delivery during surgical resection and clinically relevant adverse events.

A clinical study used ratiometric imaging methods using Raman‐encoded molecular imaging (REMI) to avoid ambiguity due to non‐specific contrast sources.^[^ ^198^ ^]^ Liu et al. used 57 fresh tumor tissues sample from 29 patients to quantify the overexpression of four cancer biomarkers, including HER2, membrane estrogen receptor (mER), EGFR, and CD44, by locally applying targeted SERS NPs at the same time (Figure 19B).^[^ ^135^ ^]^ The results demonstrated that REMI could quickly detect positive surgical margins (< 15 min), with a specificity and sensitivity of 92.1% and 89.3%, respectively. Thus, this technique could be used to guide the removal of tumors.

## 4 Summary and Perspective

SERS imaging plays a vital role in biomedical applications due to its excellent sensitivity, unique specificity, less external interference, multiplexing abilities, and integration of multiple functionalities. This technique has various applications in biomedical fields, including 1) disease diagnosis, 2) the identification of tumor edges for intraoperative guidance, 3) the direct detection of biomarker levels in complex samples and in vivo, 4) integrated SERS mapping‐guided on‐site surgery systems, 5) a SERS‐guided theranostic platform, and 6) nanotoxicity reduction and the rapid degradation of SERS tags.

This review summarized the progress of SERS as a highly sensitive and specific technology for in vivo imaging and biosensing. The detailed overview of the selection of SERS active materials, structure design, and the biocompatibility of SERS tags in in vivo imaging may facilitate the design of new SERS probes. Then, from the viewpoint of in vivo imaging and clinical translation, SERS‐based diagnostic techniques were reviewed, including targeted and non‐targeted cancer detection, the multiplexed identification of cancer, the detection of microtumors and identification of tumor margins, SERS combined with multimodal imaging, and as a multifunctional theranostic platform. SERS‐sensing based on the physiological environment was also reviewed, including the in vivo detection of biomarkers and the responsive detection of the biological microenvironment (pH, ROS, RNS, and metal ions). The progress and challenges in instrumentation and translation were also highlighted.

The development and prospects of using SERS imaging in biomedicine were also discussed and summarized. First, good SERS tags are a foundation for accurate in vivo imaging and sensing. SERS nanoprobes should exhibit extremely strong Raman signals and low toxicity. Therefore, it is necessary to rationally develop SERS active materials and apply a protective coating to improve their biocompatibility and reduce their toxicity. Although silica‐coated SERS tags are used clinically, the long‐term toxicity of SERS nanoprobes is still an obstacle in human trials. The biocompatible and well‐stabilized nanoprobes need extensive research to promote their application in human clinical practice. Second, the EPR effect is also used for cancer detection, and some studies reported that SERS‐targeted imaging exhibited higher sensitivity and specificity. Therefore, the use of controlled targeting ligands for actively targeted imaging should be further explored to improve detection accuracy. Third, as a promising tool in clinical applications, SERS also showed great prospects in disease diagnosis and treatment. Therefore, it can be used to integrate both when combined with drug release and PTT. In addition, the combination of multiple functions, such as diagnosis and treatment, helps to improve detection accuracy. The local application of SERS probes reduced toxicity when combined with a handheld Raman scanner or endoscopic probes for cancer diagnosis in clinical practice. Fourth, in terms of instruments, a number of commercially available portable Raman spectroscopy systems exhibited the advantages of convenient operation, strong portability, and fast acquisition speed, promoting their application in in vivo imaging. Previous Raman excitation mostly used NIR incident excitation that belongs to the first biological window, and NIR excitation of the second optical window provides more effective tissue penetration due to lower absorption and scattering effects. Fifth, SERS imaging needs a long acquisition time, and it is still difficult to image larger regions in clinical trials. Integrated tools capable of rapid, non‐invasive, and sensitive detection at low cost are needed in the clinical field, that is, the development of high‐resolution, fast‐imaging instruments for the real‐time identification of tumor edges.

Further work should focus on the following aspects: 1) improvements in the biocompatibility and stability of nanoprobes for human clinical trials, 2) the use of controlled targeting ligands for actively targeted imaging to improve detection accuracy, 3) SERS with handheld Raman or endoscopy for clinical trials, and 4) improvements in instruments to enhance the depth of detection and imaging speed, reducing costs to increase utilization. We hope that this review will offer some guidance in designing SERS sensors and the applications of in vivo SERS imaging.

## Conflict of Interest

The authors declare no conflict of interest.
